# Polyamine depletion inhibits murine and human norovirus infection

**DOI:** 10.1128/mbio.02033-26

**Published:** 2026-10-06

**Authors:** Maryna Chaika, Heike Laschin, Marina Pekelis, Alexander Müller, Carmen Mirabelli, Sandra Niendorf, Christiane E. Wobus, Stefan Taube

**Affiliations:** 1 University of Lübeck, Institute of Virology and Cell Biology, Lübeck, Germany; 2 Consultant Laboratory for Norovirus, Department of Infectious Diseases, Robert Koch Institute 9222, Berlin, Germany; 3 Department of Microbiology and Immunology, University of Michigan Medical School 12266, Ann Arbor, Michigan, USA; National Institutes of Health, Bethesda, Maryland, USA

**Keywords:** norovirus, polyamine, apoptosis, antiviral, human intestinal enteroid (HIE), DFMO

## Abstract

Noroviruses are non-enveloped, positive-sense RNA viruses in the family *Caliciviridae*. Human noroviruses (HNoVs) are the leading cause of viral gastrointestinal disease. Despite their clinical relevance, therapeutic strategies for norovirus infections remain limited, partly due to challenges in cultivating HNoV and limited understanding of virus-host interactions. Polyamines (PAs) are small polycationic metabolites derived from amino acid metabolism that are essential for diverse cellular functions and serve as important host factors for many RNA viruses. However, their role in norovirus infection has remained elusive. Here, we demonstrate that PAs are critical for productive infection for both HNoV and murine norovirus (MNV), underscoring a conserved and functionally significant dependency across species. Functional analysis in MNV-infected cells showed that PA depletion resulted in a marked reduction in major capsid protein synthesis and reduced viral titers following the first replication cycle, coinciding with a loss of virus-induced cytopathic effect (CPE). In PA-depleted cells, viral titers and CPE could be restored by exogenous supplementation with spermine or spermidine (metabolites of the PA biosynthetic pathway), cholesterol, or a PI3-kinase inhibitor. In addition to MNV, depletion of PAs completely ablated HNoV infection in human intestinal enteroids, which was also partially rescued by adding exogenous PAs. Collectively, these findings identify PAs as a novel host dependency factor that facilitates norovirus infection and highlight PA metabolism as a potential therapeutic target for antiviral intervention against HNoV.

**IMPORTANCE:**

Human noroviruses (HNoVs) are a major cause of acute viral gastroenteritis worldwide, yet the development of effective antivirals and vaccines has been hampered by the lack of small-animal models and robust in vitro systems to propagate the virus. Murine norovirus (MNV) shares key features with HNoV and serves as a valuable surrogate for studying norovirus biology. In this study, we identify host-derived polyamines (PAs) as essential and conserved metabolites required for productive infection for both HNoV and MNV. PAs exert multifactorial effects on norovirus infection, particularly by facilitating virus-induced apoptosis and promoting virion release in MNV-infected cells. Notably, PA depletion using difluoromethylornithine (DFMO), a well-established inhibitor of PA synthesis, effectively suppressed MNV and HNoV infection. Therefore, our results provide new insights into norovirus-host interactions and highlight the potential of a host-targeting antiviral approach to limit norovirus infection. Furthermore, our findings provide a rationale for further investigating DFMO, a licensed and well-tolerated drug, as a potential therapeutic option for chronic norovirus infections in immunocompromised patients.

## INTRODUCTION

Noroviruses are non-enveloped, positive-sense, single-stranded RNA viruses belonging to the family *Caliciviridae* (1). Human norovirus (HNoV) is a leading cause of viral gastroenteritis worldwide, characterized by symptoms ranging from acute vomiting to diarrhea (2). Although HNoV infection is typically self-limiting in healthy individuals, it can cause prolonged illness and chronic viral shedding in immunocompromised patients, with the potential for severe or even fatal outcomes (3). Despite its global health impact, no licensed antivirals or prophylactic vaccines are currently available for HNoV (4). Progress in therapeutic development has been hindered by the historical lack of suitable models to support robust HNoV replication *in vitro (5*). While histo-blood group antigens (HBGAs) have long been recognized as key host factors, they alone are not sufficient for productive infection (6). A major breakthrough in the field was the establishment of stem cell-derived human intestinal enteroid (HIE) cultures, which support HNoV replication from human stool isolates (7, 8). This system has enabled critical advances in our understanding of norovirus pathogenesis and has facilitated the first antiviral drug screens (9).

Murine norovirus (MNV), discovered in 2003, remains the only norovirus that can be efficiently and productively cultivated in cell culture (10). In immunocompetent mice, MNV-1 infection is self-limiting and typically resolves within seven days post-infection (11), whereas in immunodeficient mice, infections can become chronic or fatal (10). MNV shares numerous molecular, structural, and biological characteristics with HNoV, making it a valuable surrogate model for studying HNoV biology (12). A key distinction, however, is MNV’s use of the murine CD300lf/ld protein as a *bona fide* entry receptor (13, 14), which currently has no known functional homolog in human HNoV infection (6). Acute strains, such as MNV-1, infect myeloid-derived cells, including RAW264.7 macrophages, BV-2 microglial cells (15), as well as primary monocyte-derived cells (16). In contrast, persistent strains such as CR3 or CR6 establish long-term infection by continuously reinfecting specialized intestinal epithelial cells known as tuft cells, where they evade immune clearance through suppression of host antiviral responses (17–19).

Like other enteric viruses, MNV employs both lytic and non-lytic infections, influencing viral pathogenesis and release (20). While MNV infection generally causes a pronounced cytopathic effect (CPE) (10), it does not do so in mature murine B-cells such as M12 cells (21). This is contrary to immature murine B cells, such as WEHI-231, where the same MNV causes an acute lytic infection, highlighting that cellular factors contribute to the lytic outcome of infection.

The phosphoinositide 3-kinase (PI3K)-Akt-mTOR pathway is frequently hijacked by RNA viruses to modulate apoptosis, thereby influencing virus-induced CPE and viral release (22). MNV NS3 carries a mitochondrial localization signal and an MLKL-like membrane-disruption domain that drives cell death through mitochondrial targeting (23). During early stages of infection, MNV suppresses apoptosis, that is, via activation of the PI3K/Akt signaling pathway (20, 24). This early inhibition of apoptosis allows sufficient time for viral replication and assembly and influences the balance between intracellular retention and release of virions. In contrast, the mechanisms by which HNoV regulates apoptosis are less well understood. Similar to MNV, the HNoV non-structural protein NS3 (NTPase) induces apoptosis, as shown by caspase activation and PARP cleavage (25, 26). Other viral proteins, such as NS1/2 (N-terminal domain) and NS4 (P22), directly interact with NS3 and enhance its pro-apoptotic activity (27). This highlights that coordinated modulation of both viral and host factors is critical for controlling apoptosis, CPE, and the balance between viral replication and release.

**Fig 1:**
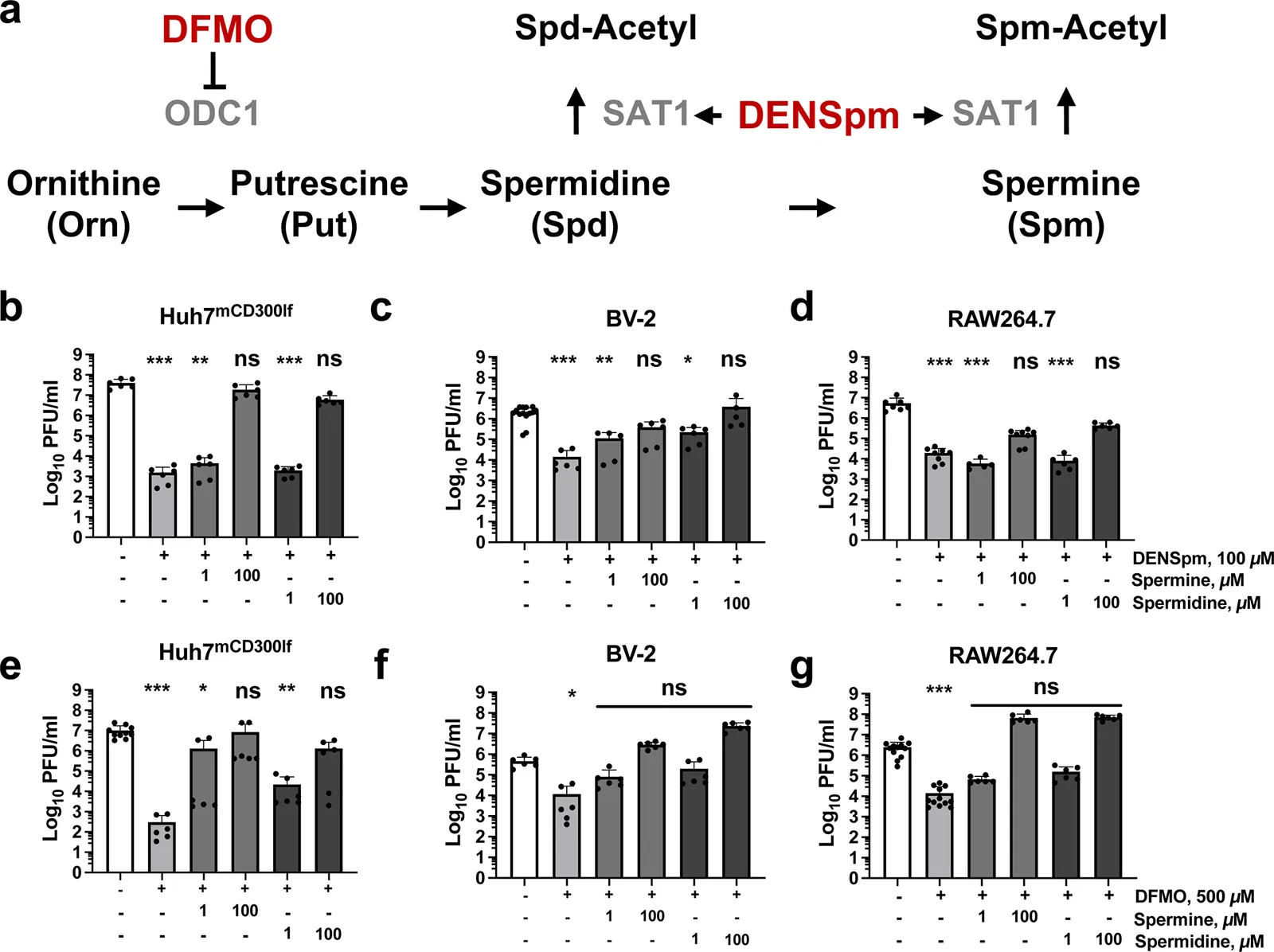
Polyamine (PA) depletion restricts MNV infection in immune and epithelial cells. Polyamines (PAs) biosynthesis and catabolic pathway (**a**). Huh7^mCD300lf^ (**b and e**), BV-2 (**c and f**), and RAW264.7 (**d and g**) were pretreated with 100 μM DENSpm for 24 h (**b–d**) or 500 μM DFMO for 96 h (**e–g**). Cells were inoculated with MNV-1 at an MOI 0.1 and incubated for 1 h at 4°C. The inoculum was removed and replaced with culture medium (−) or culture medium supplemented with DENSpm, DFMO with or without 1 or 100 μM of spermine or spermidine. Samples were lysed 24 h post-infection (hpi), and virus titers were determined by plaque assay. Data represent the means ± standard deviation (SD) of three independent biological assays with two technical replicates. Individual data points are shown. Statistical significance was assessed using the Kruskal–Wallis test compared to non-treated/infected: ns, not significant; *P ≤ 0.05; **P ≤ 0.01; and ***P ≤ 0.001.

Viruses rely on host factors to complete their infection cycle; among these, metabolites such as polyamines (PAs) have emerged as essential regulators of viral replication and pathogenesis (28, 29). PAs play a crucial role in maintaining intestinal barrier integrity by regulating epithelial cell proliferation, migration, and tight junction stability (30). PAs are also crucial for cellular processes such as cell cycling, nucleic acid binding, apoptosis regulation, and membrane fluidity (31–35). PAs are also involved in translation regulation, through hypusination, where PAs are covalently attached to the eukaryotic translation initiation factor eIF5A (36). Hypusination then promotes the expression of cholesterol synthesis genes by supporting SREBP2 synthesis. Mammalian cells exclusively synthesize biogenic PAs, for example, putrescine, spermidine, and spermine, from an ornithine precursor through ornithine decarboxylase (ODC1) (28) (Fig. 1a). PA depletion is associated with decreased expression of caspases and pro-apoptotic Bax/Bad proteins (37) and leads to cell survival upon stimulation of intestinal epithelial cells with extrinsic apoptotic signals (38).

PAs are known as host factors for a broad range of RNA and DNA viruses (28, 29), by playing a role at different steps of viral infection. For example, coxsackievirus B3 (CVB3), a picornavirus, requires PA for cell entry (36), while Zika virus (ZV) and other flaviviruses require PAs for genome replication (39, 40). Bunyaviruses, like Rift Valley fever virus (RVFV), incorporate PAs into virions and rely on PAs for production of infectious particles (41, 42).

Here, we investigate the role of PA in MNV and HNoV infections and their contribution to capsid synthesis, virus-induced CPE, and PI3K- and cholesterol-dependent pathways.

## MATERIALS AND METHODS

### Cell culture

Murine microglial cell line BV-2, murine macrophage cell line RAW264.7, and recombinant human hepatoma cell line Huh7, stably expressing murine CD300lf (Huh7^mCD300lf^) (43) were used. BV-2 cells were maintained in DMEM (Dulbecco’s modified Eagle medium) supplemented with 5% fetal calf serum (FCS), 1% penicillin/streptomycin (P/S) (Capricorn Scientific), 1% l-glutamine (l-Glut) (C-C-Pro), and 1% non-essential amino acids (NEAAs) (Capricorn Scientific). RAW264.7 and Huh7^mCD300lf^ were maintained in DMEM supplemented with 10% FCS, 1% P/S, 1% l-Glut, and 1% NEAA. For passaging, RAW264.7 and BV-2 cells were detached by scraping, whereas Huh7^mCD300lf^ cells were detached by trypsinization using 0.05% Trypsin-EDTA (Thermo Fisher Scientific). All cells were incubated at 37°C, 5% CO_2_, 95% relative humidity (RH).

### Preparation of MNV virus stocks

The MNV-1 (GV/MNV1/2002/USA) CW1 isolate was generated by reverse genetics with minimal passaging (43) and used up to passage 4. Wild-type CR3 (GV/CR3/2005/USA) was used up to passage 4 since plaque isolation. Virus stocks were prepared by infecting BV-2 cells at a multiplicity of infection (MOI) ~0.1 until strong cytopathic effect was visible, usually ~24 h post-infection (hpi). Flasks were freeze-thawed two times and lysate centrifuged at 4,500 × *g* for 10 min at 4°C to remove cell debris. The clarified supernatant was precipitated by 40% ammonium sulfate, overnight, rotating at 4°C. The precipitated virus was pelleted by centrifugation at 10,000 × *g* for 15 min at 4°C. Virus was resuspended in 100 mM sodium phosphate buffer, pH 7.4, sterile-filtered (0.22 µm pore size). Titered aliquots were stored at −80°C.

### Chemical inhibitors

Difluoromethylornithine hydrochloride hydrate (DFMO, also known as Eflornithine) (Tebu-Bio, #T2593), and N1, N11-diethylnorspermine tetrahydrochloride (DENSpm) (Bio-Techne, #0468) were prepared in ddH_2_O as 50 and 2.5 mM stocks, respectively, and stored in aliquots at −20°C. LY294002 (LY) hydrochloride (Tebu-Bio, #T4079) was prepared in dimethyl sulfoxide (DMSO) (Carl Roth, #A994.2) as 5 mM stocks and stored in aliquots at −20°C. Staurosporine (BioMol, #LKT-S7600) was prepared in DMSO as 1 mM stocks and stored in aliquots at −20°C. Spermine (Cayman Chemicals, #18041) and spermidine (Cayman Chemicals, #14918) were prepared as 1 mM stocks in DMEM supplemented with 1% P/S and 1% l-Glut, and stored short term at 4°C.

### PA depletion/degradation and MNV infection

For PA depletion, cells were treated with 500 µM DFMO at ~60–70% confluency for 4 days. At day 3, cells were re-seeded in 24-well tissue culture plates at 4 × 10^5^ cells/well for RAW264.7 and at 2 × 10^5^ cells/well for BV-2 and Huh7^mCD300lf^ cells and freshly supplemented with DFMO-containing medium for the final day of treatment. For PA degradation, cells were treated with DENSpm at 100 µM after plating and incubated overnight for an 18 h treatment. For MNV infections, cells were inoculated with MNV diluted in cold PBS at the indicated MOI and incubated for 1 h on ice. Virus inoculum was removed, and cells were washed three times with ice-cold PBS, after which warm culture medium plus the indicated supplements and incubated at 37°C in 5% CO_2_ at 95% RT as indicated. After incubation, cells and/or supernatants were lysed by freeze-thawing twice and titered as indicated, either by plaque assay or endpoint dilution. For protein analysis by sodium dodecyl sulfate (SDS)-polyacrylamide gel electrophoresis (PAGE) and western blotting, RAW264.7 cells were seeded in six-well cell culture plates at 1 × 10^6^ cell/well before infection the following day as described above. At the experimental endpoint, the supernatant was removed, and cells were collected in an ice-cold lysis buffer (PBS, 100 mM NaCl, 1% Triton-X, pH 8.0) before protein extraction.

### Plaque assay

Infectious virus titers were measured using plaque assay as previously described (44). Briefly, BV-2, RAW264.7, or Huh7^mCD300lf^ cells (43) were plated in six-well tissue culture plates 1 day prior to infection at 5 × 10^5^, 5 × 10^6^, or 2 × 10^5^ cells/well, respectively. As BV-2 cells were less adherent to our cell culture plastic, endpoint dilutions were the preferred method for this titration. Infectious samples were serially diluted 1:10 in a total volume of 1.25 mL (1,125 µL PBS or sodium phosphate buffer, 125 µL sample). Cell culture medium was aspirated, and cells were infected in duplicate with 500 µL of the respective diluted virus per well. Inoculated plates were incubated at 37°C, 5% CO_2_, 95% RH for 1 h. Virus was aspirated and replaced with an overlay medium (1.2% Avicel [IMCD Deutschland GmbH, #RC-581NF], 1× MEM [Thermo Fisher Scientific, #61100087], 5% FCS; 1% P/S, and 1% l-Glut). Plates were incubated at 37°C, 5% CO_2_, 95% RH for 48 h. The overlay medium was aspirated; cells were washed with PBS and stained with 1 mg/mL erythrosine B (Sigma-Aldrich, #198269) in PBS.

### Endpoint titration determining tissue culture infectious dose 50 (TCID_50_)

Infectious virus titers were measured by endpoint titration (45), as an alternative to the preferred plaque assay. BV-2 cells were the preferred cell line, as only 1 × 10^4^ cells/96-well plate were needed, and no trypsinization was required. Cells were seeded in flat-bottom 96-well tissue culture plates and used immediately after cell adherence. Virus-containing samples were serially diluted using 180 μL medium and 20 μL virus sample. Plates were incubated for 3–4 days at 37°C, 5% CO_2_, and 95% RH. After incubation, wells were scored for CPE by light microscopy. Viral titers were calculated as TCID_50_/mL using the Reed–Muench method (46, 47).

### RT-qPCR-based MNV-1 attachment assay

Mock- or DFMO-treated Huh7^mCD300lf^, BV-2, or RAW264.7 cells were seeded 1 day prior to infection in six-well tissue culture plates (2 × 10^5^, 5 × 10^5^, and 5 × 10^6^ cells/well, respectively). The following day, virus inoculum was prepared to an MOI of 5 by diluting MNV-1.CW1 stock in PBS (pH 7.4) supplemented with spermine and/or DFMO. A total of 500 μL inoculum was added to each well and incubated on ice for 1 h to allow virus attachment. Following incubation, the inoculum was aspirated, and cells were washed three times with ice-cold PBS (pH 7.4) to remove unbound virus. After the final wash, wells were aspirated completely, and cell monolayers were lysed in 500 μL TRIzol Reagent (Zymo Research Europe GmbH). Genome equivalents (GEs) were determined as previously described (43) and normalized to the total amount of RNA extracted. Briefly, total RNA was extracted using the Direct-zol RNA MiniPrep kit (Zymo Research Europe GmbH) according to the manufacturer’s instructions. RNA concentration was determined using a NanoDrop 2000 spectrophotometer (Thermo Fisher Scientific), assessing A260/280 and A260/230 ratios. GEs were determined using the Luna Universal Probe One-Step RT-qPCR Kit (NEB) with primers and a probe targeting the NS1/2 in ORF1 (Forward: 5′-GTGCGCAACACAGAGAAACG; Reverse: 5′-CGGGCTGAGCTTCCTGC; Probe: 5′-[6-FAM]-CTAGTGTCTCCTTTGGAGCACCTA-[BHQ1]). A synthetic dsDNA fragment (gBlock, Integrated DNA Technologies [IDT]) corresponding to the amplified cDNA was used to generate a standard curve for quantification of genome eq.

### Antibodies

Antibodies against PARP (Cell Signaling Technologies, #9542T) were diluted 1:1,000 for protein detection during western blotting. Beta-actin (Bio-Techne, #MAB8929-SP) was used at a final concentration of 0.02 μg/mL. The broadly specific norovirus VP1-specific monoclonal antibody TV19 (48) was used at a 1:2,000 dilution (kindly provided by Peter Sander, R-Biopharm AG, Germany). Secondary antibodies goat anti-rabbit HRP-IgG (Dianova, #111-045-003) and goat anti-mouse (Dianova, #111-035-003) were diluted 1:3,000 during western blotting.

### Protein extraction

Cells were scraped in lysis buffer (PBS, 100 mM NaCl, 1% Triton-X, pH 8.0) from wells using a sterile cell scraper and transferred into sterile centrifuge tubes. To ensure maximum cell lysis, tubes were incubated on ice for 10–15 min. Lysed cells were centrifuged at 4°C, 16,000 × *g* for 20 min. The cleared lysate was transferred into fresh tubes and mixed (1:2) with sample loading buffer (62.5 mM Tris, 10% glycerol, 5% beta-mercaptoethanol [BME], 2% SDS, and 0.1% bromophenol in ddH_2_O, pH 6.8) and stored at −80°C in aliquots.

### Spermidine detection via indirect ELISA

Human spermidine GENLISA ELISA Kit (Krishgen BioSystems, #KBH22460) was utilized for spermidine quantification. RAW264.7 macrophages were pretreated with DFMO, DENSpm, spermine, and spermidine as mentioned above. Cells were collected into a fresh tube at 5 × 10^5^ cells/condition and pelleted by centrifugation for 5 min at 500 × *g*. Cell culture medium was aspirated, and cells were resuspended in 200 µL PBS. Intracellular contents were released into PBS by three freeze-thaw cycles. Cellular debris was removed by centrifugation for 10 min at 800 × *g*. Samples or standard dilutions were added in a 100 µL volume to the pre-coated wells with anti-Spd antibodies. The samples were incubated for 80 min at 37°C, then washed four times. Biotinylated anti-spermidine antibody solution was added to samples according to the manufacturer’s protocol and incubated for 50 min at 37°C. After four washes, streptavidin-HRP conjugate solution was added to the samples and incubated for 50 min at 37°C. After another four washes, TMB substrate was incubated with samples for 10 min at 37°C and inactivated with stop-solution. TMB substrate conversions were measured at 450 nm with a microplate reader (Tecan Infinite M Plex). Spermidine concentration in experimental samples was calculated using the standard curve.

### Trypan blue cell viability assay

Cell density was determined using a hemacytometer. Ten microliters of a 0.4% trypan blue solution (Thermo Fisher Scientific, #15250061) was added to 10 μL of cell suspension. The number of viable cells was determined according to the manufacturer’s recommendations.

### WST-1 cell metabolic assay

WST-1 (Roche, #5015944001) cell proliferation assay was used to assess cell viability after drug treatments. Briefly, cells were treated and/or supplemented as described for the assay. WST-1 reagent was added at a 1:10 dilution to cell culture medium in 24-well plates and incubated at 37°C, 5% CO_2_, and 95% RH. Conversion of WST-1 to formazan was evaluated every 15 min by measuring the absorbance at 450 nm and for reference at 660 nm. Measurements were performed until control samples reached an optical density (OD) of 1–1.2 on a microplate reader (Tecan Infinite M Plex). Data were normalized according to the manufacturer’s recommendations and compared as indicated.

### SDS-PAGE and western blotting

Tricine-sodium dodecyl sulfate (SDS)-polyacrylamide gel electrophoresis (PAGE) was used (49). Briefly, frozen protein lysates were thawed on ice and denatured at 90°C for 10 min on a heat block. Lysed samples were centrifuged (5 min, 15,700× *g* and 4°C) before loading onto gels. Proteins of interest up to 70 kDa were separated in 10% Tricine-SDS-PAGE gels, while 12% gels were used for larger proteins. SDS-PAGE was conducted at 80 V for 10 min, followed by 120 V for 50 min (10% gels), 120 V for 65 min (12% gels). Proteins were transferred onto a 0.2 µm nitrocellulose membrane (Omnilab) in transfer buffer (25 mM Tris, 193 mM glycine, and 20% methanol in ddH_2_O) at 100 V (60 min for 10% gels or 90 min for 12% gels). Membranes were blocked with 5% fat-free milk (Carl Roth) in PBS + 0.05% Tween 20 (PBS-T) overnight at 4°C. The following steps were conducted at RT. After blocking, membranes were incubated with the respective primary antibody diluted in 5% fat-free milk in PBS-T for 1 h, followed by three washes with PBS-T for 5 min each. Membranes were then incubated with the appropriate secondary antibody diluted in 5% fat-free milk in PBS-T for 1 h and subsequently washed three times with PBS-T for 5 min each. Protein signals were detected using enhanced chemiluminescence (ECL) substrate (Pierce ECL Western Blotting Substrate, Thermo Fisher Scientific, #32209, or Western Lightning Plus-ECL, PerkinElmer, #NEL103001). Signals were visualized using the ImageQuant 800 Western Blot Imaging System (Amersham).

### HNoV stool samples

HNoV isolates were obtained from stool samples from norovirus positive patients at the University Clinic of Schleswig-Holstein (UKSH). HNoV was genotyped by phylogenetic analysis of ORF1 and ORF2 sequences as previously described (50). HNoV GEs were determined by RT-qPCR as previously described (51, 52). Short-term stocks were prepared from stool filtrates (solution in Opti-MEM, filtered through 0.22 µm membrane) and stored as aliquots at −80°C. HNoV inoculum was freshly prepared as indicated and directly used for infection of HIE.

### HIE maintenance and infection with HNoV

HIE line HT-124 (52) was maintained at 37°C, 5% CO_2_, 95% RH in basal membrane extract (BME, Matrigel Matrix, Corning, #354234) with growth medium (IntestiCult, STEMCELL Technologies, #06010). HIEs were passaged every 7 days with a 1:2 split ratio and medium replacement every other day. Differentiation was by Wnt3a withdrawal, switching to standard differentiation medium (Advanced DMEM-F12, 1% P/S, 1% HEPES, 5% Noggin conditioned medium, B-27 [Invitrogen, #17504-044], N-2 [Invitrogen, #17502-048], 50 ng/mL murine EGF [Thermo Fisher Scientific, #PMG8043], 1 mM N-acetyl cysteine [Sigma-Aldrich, #A9165], 10 nM Leu15-Gastrin [Sigma-Aldrich, #G9145], and 500 nM A 83-01 [Tocris, #2939]) or putrescine-free differentiation medium (Advanced DMEM-F12, 1% P/S, 1% HEPES, 5% Noggin conditioned medium, 50 ng/mL murine EGF [Thermo Fisher Scientific, #PMG8043], 1 mM N-acetyl cysteine [Sigma-Aldrich, #A9165], 10 nM Leu15-Gastrin [Sigma-Aldrich, #G9145], and 500 nM A 83-01 [Tocris, #2939]) for 7 days with medium replacement every other day.

On the day of infection, 3D-HIEs were collected in washing medium (Advanced DMEM-F12, 1% P/S, and 1% HEPES) by scraping and pelleted at 90 × *g* for 4 min at 4°C. HIEs were resuspended in differentiation medium and transferred to 1.5 mL tubes. HNoV filtrates were added at an estimated MOI of 1 (based on GEs) to HIEs with 500 µM bile acid (glycochenodeoxycholic acid, GCDCA, Sigma-Aldrich, #G0759). Each sample contained an estimated 40–60 matured spheres in a final volume of 200 µL/tube. Viruses and HIEs were incubated for 1 h at 37°C with occasional mixing. HIEs were washed three times with washing medium and pelleted (100 × *g* for 2 min at 4°C). Spheres were plated in 30 µL/sample of BME into a pre-warmed 24-well plate. Plates were left for 10 min at 37°C to ensure polymerization of the BME. HIEs were supplemented with differentiation medium including 2 µM of Ruxolitinib (Sigma-Aldrich, #AMBH324A54EE) and 500 µM bile acid GCDCA. HIEs were harvested in 400 µL/well of TRIzol reagent (Zymo Research Europe GmbH), immediately (0 hpi) or at 48 hpi.

### Titration of HNoV

HNoV titers were determined as previously described (43, 52). Briefly, total RNA was extracted from samples using the Direct-zol RNA MiniPrep kit (Zymo Research Europe GmbH, #R2073). HNoV GEs were determined using the Luna Universal Probe One-Step RT-qPCR Kit (NEB) with specific primers and probe targeting the ORF-1/ORF-2 junction (forward, AGCCAATGTTCAGATGGATG; reverse, TCGACGCCATCTTCATTCAC; and TaqMan probe, TGGGAGGGCGATCGCAATCTGGC). A synthetic dsDNA fragment (gBlock, Integrated DNA Technologies [IDT]) corresponding to the amplified cDNA was used to generate a standard curve for quantification of HNoV GEs.

### Statistical analysis

Graphs were generated and statistically analyzed using GraphPad Prism, version 10 (GraphPad Software Inc., La Jolla, CA, USA). For comparisons among multiple groups, a parametric ANOVA was applied when data met normality assumptions. The non-parametric Kruskal–Wallis test was used when normality could not be confirmed using the Shapiro–Wilk test.

## RESULTS

### MNV is sensitive to PA depletion

PAs are required for a number of distinct viral families, including enteric viruses, and other RNA viruses (29). To examine the role of PAs in norovirus infection, we sought to determine MNV sensitivity to PA depletion (Fig. 1) using well-established PA inhibitors (53).

First, we employed N^1^,N^11^-diethylnorspermine (DENSpm), an activator of spermine/spermidine N^1^-acetyltransferase 1 (SAT1). SAT1 catalyzes the acetylation of spermine and spermidine, promoting their degradation and excretion, thereby depleting them from the cell (Fig. 1a). Three different cell lines were used: Huh7^mCD300lf^, which constitutes a recombinant epithelial cell expressing the murine mCD300lf receptor for susceptibility (43), BV-2, and RAW264.7, which are microglia-like and macrophage-like monocyte-derived immune cell lines. All cells were pretreated with 100 µM DENSpm for 24 h prior to infection. Following pretreatment, cells were infected with MNV-1 at an MOI of 0.1 for 1 h on ice, then shifted to 37°C in the continued presence of DENSpm, with and without supplemented spermine or spermidine. Viral titers were then quantified by plaque assay at 24 hpi. The data showed that DENSpm treatment significantly impaired MNV-1 infection across all tested cell lines (Fig. 1b through d), with significant reductions of 2–3 logs in BV-2 and RAW264.7 cells, and up to 5 logs in Huh7^mCD300lf^ cells. Notably, supplementation with 100 µM of either spermine or spermidine restored viral titers close to untreated controls (Fig. 1b through d), which is interesting as DENSpm activates the catabolism of either of these molecules.

Second, we employed difluoromethylornithine (DFMO), a well-tolerated and specific inhibitor of ornithine decarboxylase 1 (ODC1) (Fig. 1a). DFMO blocks the rate-limiting step in PA biosynthesis, the conversion of ornithine to putrescine, resulting in reduced levels of putrescine, spermidine, and spermine. Huh7^mCD300lf^, BV-2, and RAW264.7 cells were treated with 500 µM DFMO for 4 days prior to infection (Fig. 1e through g). Cells were inoculated with MNV-1 at a multiplicity of infection (MOI) of 0.1 for 1 h on ice, followed by incubation for 24 h at 37°C in the continued presence of DFMO, with or without supplementation of spermine or spermidine. Similar to DENSpm, DFMO treatment led to significant inhibition of MNV infection across all tested cell lines by 24 hpi (Fig. 1e through g), highlighting the role of PAs as critical host factors for MNV infection in both immune and epithelial cell types.

To support the selected concentrations for both DENSpm and DFMO, dose-response experiments were performed in RAW264.7 cells, demonstrating a concentration-dependent inhibition of MNV infection (Fig. S1).

Similar data were also obtained for the MNV strain CR3 in Huh7^mCD300lf^ cells (Fig. S2), showing that this is not restricted to MNV-1. Metabolic activity and cell viability were assessed using a WST-1 and a trypan blue exclusion assay and confirmed no negative effect on cell health during PA depletion (Fig. S3 for RAW264.7 cells). Notably, the addition of spermine or spermidine to untreated (PA-intact) cells did not alter viral titers (Fig. S4), suggesting that baseline PA levels in standard culture conditions were adequate to support infection. Supplementation with spermine or spermidine rescued viral replication in PA-depleted cells (Fig. 1e and f). Although the specificity and working concentrations of DENSpm and DFMO are well established (29), we confirmed a decrease in intracellular spermidine upon treatment in RAW264.7 cells with DFMO and DENSpm, as well as the restoration of intracellular spermidine upon supplementation in the cell culture medium (Fig. S5). While serum was postulated to contain PAs and PA-catabolizing enzymes (54), we detected no spermidine in our serum and observed normalization of spermidine levels in PA-restricted cells, indicating no negative impact on exogenous supplemented PAs in the presence of serum. While it is possible that levels were below the detection limit of the ELISA, or our cell culture serum contains PAs other than spermidine that we are unable to detect with this assay, they are unlikely to significantly impact our assay conditions.

In conclusion, our data demonstrate that PA depletion significantly blocks MNV infection across multiple cell types in a reversible and strain-independent manner.

### MNV attachment to cells is not affected by PA depletion

**Fig 2:**
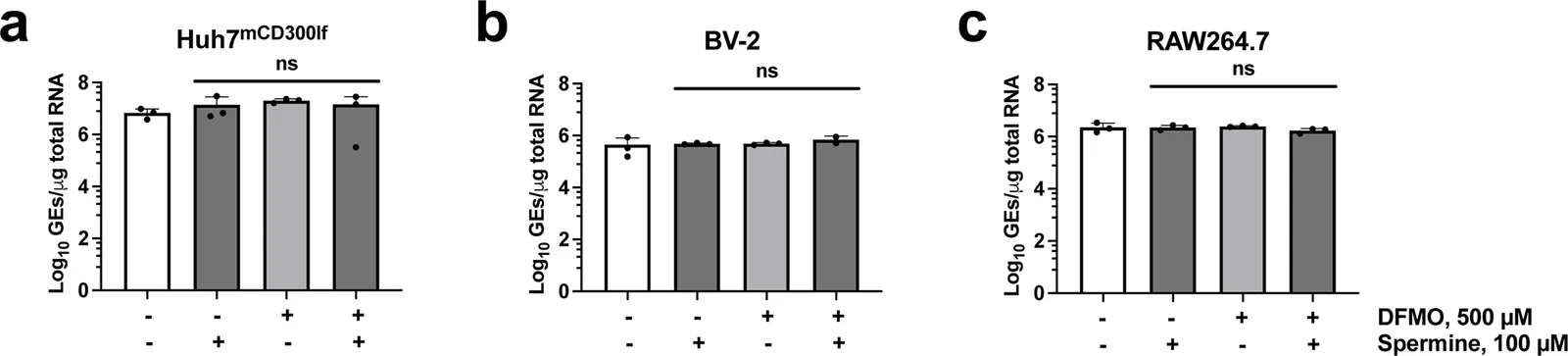
PA depletion does not alter MNV-1 attachment to cells. Huh7^mCD300lf^ (**a**), BV-2 (**b**), and RAW264.7 (**c**) were pretreated with 500 μM DFMO for 4 days. Cells were inoculated with MNV-1 at an MOI of 5 on ice in the presence of DFMO and 100 µM spermine. After 1 h of incubation to allow for virus attachment, cells were washed and harvested for total RNA extraction. MNV titers were quantified by RT-qPCR. Data represent the means ± standard deviation (SD) of three independent biological assays. Individual data points are shown. Statistical significance was assessed using the Kruskal–Wallis test and shown compared to non-treated/infected: ns, not significant.

Previous reports have shown that other enteric RNA viruses rely on PAs for efficient binding and entry into host cells (55, 56). PAs regulate the expression of the transcription factor SREBP2, which is required for intracellular cholesterol biosynthesis (36). Reduced SREBP2 activity leads to decreased cholesterol content in cellular membranes, which could affect viral entry. Since MNV entry is cholesterol-dependent (57, 58), we investigated whether PA depletion affects MNV-1 attachment to host cells. Huh7^mCD300lf^, BV-2 cells, and RAW264.7 cells were cultured in the presence of 500 µM DFMO for 4 days to deplete PAs (Fig. 2). Cells were inoculated with MNV-1 at an MOI of 5 and incubated on ice for 1 h to allow viral binding without internalization. The unbound virus was removed by washing, and cells were harvested in TRIzol for total RNA extraction. Viral GEs were quantified by RT-qPCR. MNV-1 attachment was unaffected by either PA depletion or spermine supplementation in all the tested cell lines, indicating that PA availability does not influence MNV-1 attachment.

### PA depletion does not impact MNV-1 genome levels

**Fig 3:**
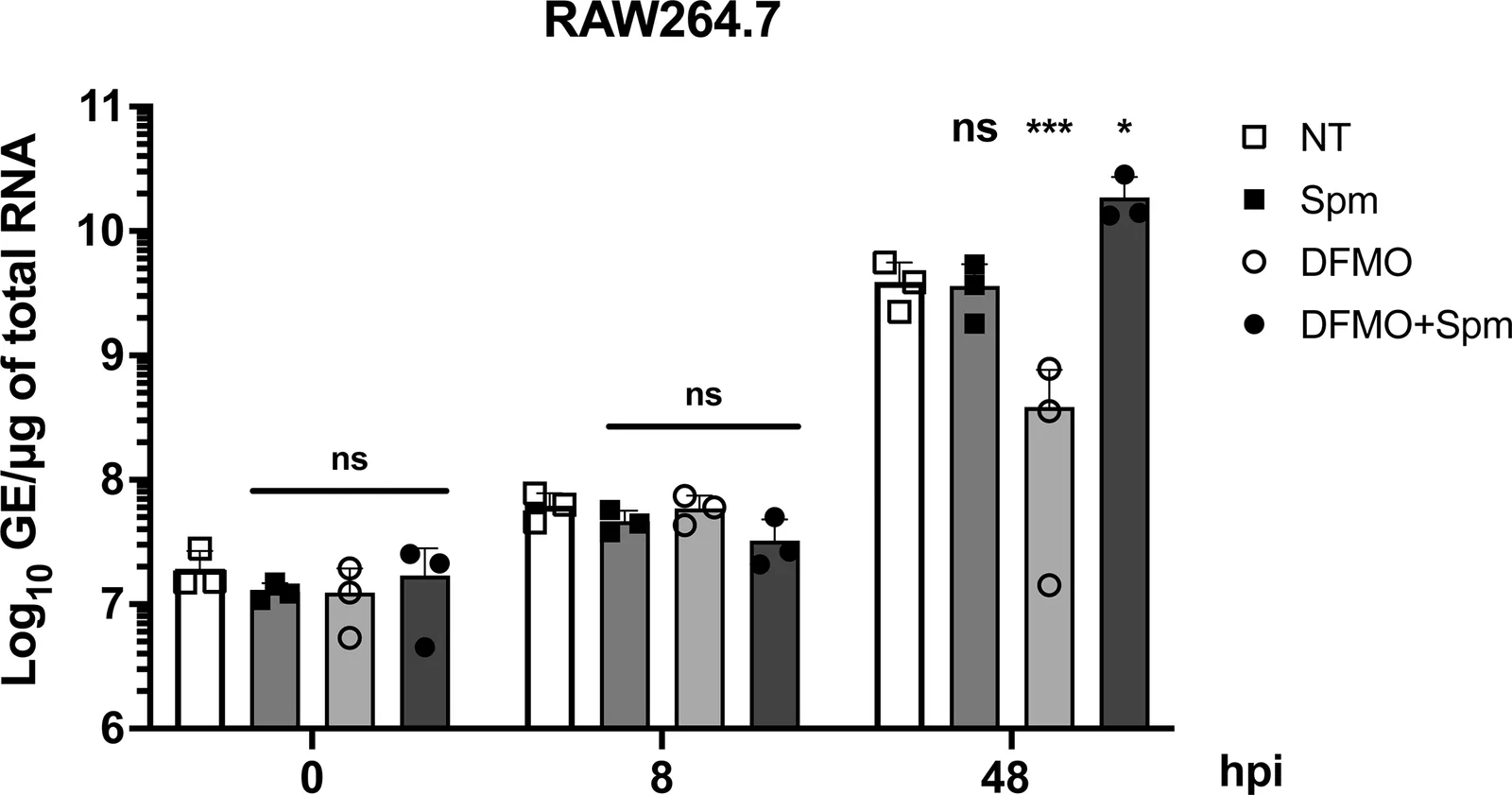
Impact of PA depletion on genome levels in RAW264.7 cells. RAW264.7 cells were treated with 500 μM DFMO for 4 days to deplete PAs, then infected with MNV-1 at an MOI of 50. Inoculated cells were supplemented with DFMO and/or 100 µM Spm. Cells were harvested by freezing at 0, 8, and 48 hpi for quantification of viral genome via RT-qPCR. Data represent the means ± standard deviation (SD) of at least three independent biological assays with two technical replicates for the plaque assay. Individual data points are shown. Statistical significance was assessed using the one-way ANOVA test and shown compared to the infected condition within the time point: ns, not significant; *P ≤ 0.05; ***P ≤ 0.001.

For several flaviviruses, including Zika and chikungunya virus, PAs are important for RNA genome replication and packaging and have been shown to promote the formation of membrane-associated replication complexes (59, 60). To investigate the effect of PAs on MNV-1 genome replication, RAW264.7 cells were pretreated with DFMO for 4 days and infected with MNV-1 at an MOI of 50 (Fig. 3). This high MOI was chosen to ensure that genome replication was measured during the initial round of infection while minimizing contributions from secondary infection, as previous studies demonstrated that lower MOIs were insufficient to infect the majority of cells synchronously. Lysates were then harvested at 0, 8, and 48 hpi, with GEs being quantified by RT-qPCR.

No significant change in genome copies was observed during attachment and the first round of infection (8 h at an MOI of 50), while a significant reduction in MNV genomes was observed at 48 hpi, suggesting that PA depletion acts downstream of genome replication.

### PA depletion prevents multiple rounds of infection

**Fig 4:**
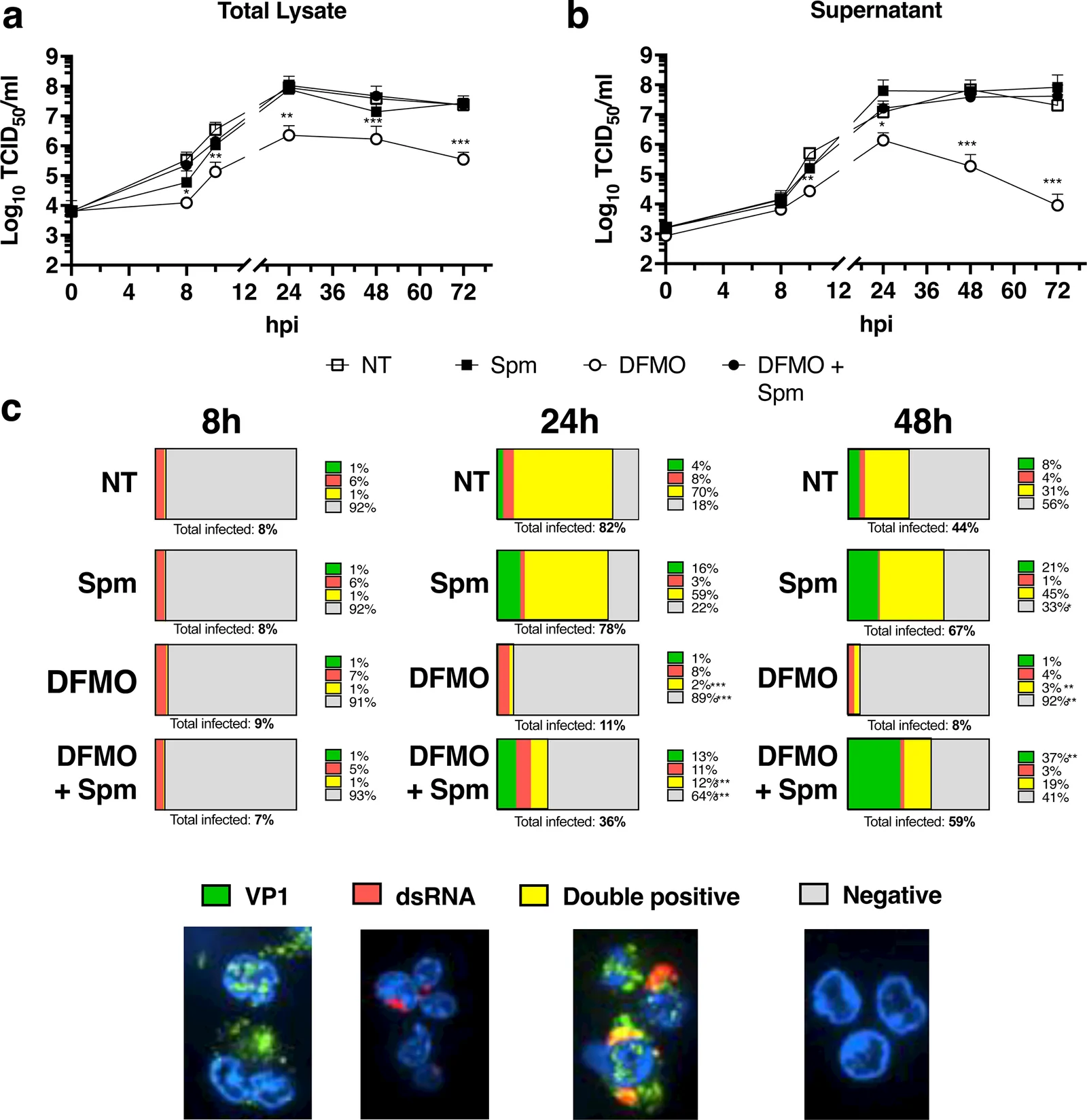
PA depletion inhibits MNV-1 replication and release. RAW264.7 cells were treated with 500 μM DFMO for 4 days to deplete PAs, then infected with MNV-1 at an MOI of 50. Inoculated cells were supplemented with DFMO and/or 100 µM Spm. Total cell lysates (cells and supernatant) (**a**) or clarified supernatant (**b**) were harvested by freezing at 0, 8, 10, 24, 48, and 72 hpi for quantification of infectious particles via TCID_50_ assay. (**c**) Cells were harvested by fixation with 4% PFA at 8, 24, and 48 hpi, stained with anti-MNV capsid rabbit serum and anti-dsRNA J2 clone. Nuclei were counterstained with Hoechst. A randomly selected field of view was analyzed per assay from three independent biological assays. Data are presented as means, and statistical significance was determined using an ANOVA relative to non-treated (NT) controls within each time point (**a and b**) or compared to non-treated/infected condition within the time point (**c**): ns, not significant (not shown for clarity); *P ≤ 0.05; **P ≤ 0.01; and ***P ≤ 0.001.

To better define the stage of infection at which PA depletion affects MNV-1 replication, infection kinetics were analyzed in RAW264.7 murine macrophages (Fig. 4). Cells were depleted of PAs and subsequently infected with MNV-1 at an MOI of 50 to achieve near-synchronous infection. Viral titers were quantified at 8–72 hpi, and infectious titers were determined from total lysates (cells and supernatant) as well as clarified supernatants to assess viral release (Fig. 4a and b).

At high MOI, MNV-1 infection was significantly reduced in both lysate and supernatant of DFMO-treated cells at all time points (8–72 hpi) and was fully restored by the addition of spermine. Notably, even at 8 hpi, the time required for MNV to complete a single round of infection, infectious titers were already significantly lower under PA depletion, despite no detectable change in genome replication at this time point as shown above (Fig. 3). Together, this suggests that PA depletion acts downstream of RNA replication.

Next, we investigated the proportion of infected cells by monitoring capsid protein (VP1) and viral dsRNA using immunofluorescence microscopy (Fig. 4c). At 8 hpi, only 7–9% of cells were positive for infection (5–7% for dsRNA, ~1% for VP1, and ~1% for both) across all conditions, despite the high MOI. Importantly, at this early time point, the proportion of infected cells did not differ between PA-competent and PA-depleted cells. Consistent with viral titers, the proportion of VP1-positive and double-positive cells increased markedly in non-treated and spermine-supplemented cells until 24 hpi, reaching up to 75%, while a steady 3–11% of cells remained dsRNA-positive only. In contrast to the PA-competent conditions, DFMO-treated macrophages showed no increase in the number of infected cells (8–11%) at any time point, indicating that no new cells were infected beyond 8 hpi, reflecting a block after the first round of infection.

In PA-depleted cells supplemented with spermine, the overall number of infected cells remained significantly lower than in non-treated controls; however, partial rescue of VP1 accumulation and an increase in double-positive cells was observed, sufficient to fully restore virus titers as observed above.

**Fig 5:**
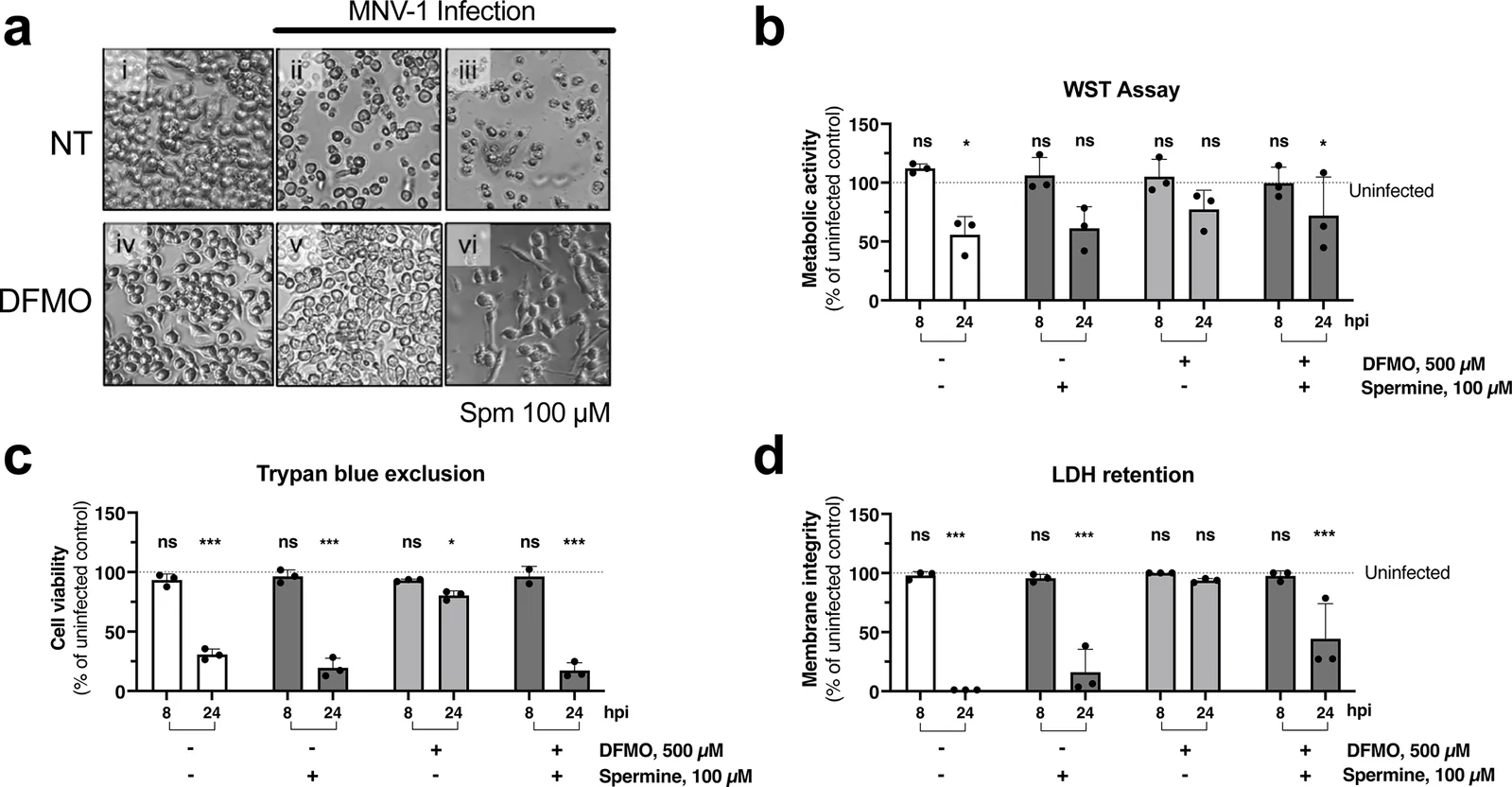
Loss of virus-induced cytopathic effect (CPE) in PA-depleted murine macrophages. RAW264.7 cells were pretreated with 500 μM DFMO for 4 days, and subsequently infected with MNV-1 at an MOI of 50 on ice for 1 h. After infection, the inoculum was removed, and cells were reconstituted with medium containing DFMO and/or 100 μM spermidine. MNV-induced CPE was analyzed 24 hpi by bright-field microscopy (**a**); cell viability was determined by WST-1 assay (**b**), trypan blue exclusion assay (**c**), and LDH retention assay (**d**) at 8 and 24 hpi and shown as percent viable compared to the respective uninfected control. Data are presented as means ± standard deviation (SD) from three independent biological experiments. Statistical significance was assessed using a one-way ANOVA and shown compared to the corresponding non-treated (NT)/non-infected sample: ns, not significant (not shown for clarity); *P ≤ 0.05 and ***P ≤ 0.001.

At 48 hpi, the overall cell numbers declined due to the CPE except for DFMO-treated cells, which showed no visible CPE (see Fig. 5). Compared to 24 hpi, the proportions of VP1-, dsRNA-, and double-positive cells remained stable in the non-DFMO treated cells. In DFMO-treated cells, no increase in the number of infected cells was observed. In the spermine rescue, infected cells increased from 36% to 59%, which was comparable to the non-DFMO treated cells. Interestingly, VP1-positive cells increased from 13% to 37%, while double-positive cells only increased from 12% to 19%, suggesting that VP1 expression precedes efficient dsRNA synthesis.

In summary, these data show that PA depletion blocks multiple rounds of MNV-1 infection in RAW264.7 cells. Despite the high MOI, DFMO-treated cells exhibited significantly reduced viral titers in both cell lysates and supernatants at all time points. Early viral RNA replication and the proportion of infected cells at 8 hpi were unaffected, indicating that the block occurs downstream of RNA synthesis. Over time, the numbers of VP1-positive and double-positive cells increased in untreated and spermine-supplemented cells, whereas DFMO-treated cells showed no further spread, and supernatant titers dropped sharply after 24 hpi, suggesting reduced virion stability. Spermine supplementation in DFMO-treated cells partially restored VP1 expression and dsRNA synthesis, resulting in viral titers comparable to those in untreated cells.

### PA restriction prevents MNV-induced cell death

During our study, we observed an almost complete loss of the CPE in MNV-infected, PA-depleted cells, prompting further investigation of this phenotype (Fig. 5). Interestingly, even at an MOI of 50 at 24 hpi, DFMO-treated cells showed no CPE (Fig. 5a, panel v) compared to infected but non-treated cells (panel ii). This phenotype was completely reversed by adding spermidine (panel vi) compared to infected and DFMO-treated (panel v).

To assess cellular viability, we quantified metabolic activity of the culture using a WST-1 assay (Fig. 5b) and evaluated membrane integrity using the LDH retention assay (Fig. 5d) and trypan blue exclusion assay (Fig. 5c) at 8 and 24 hpi. At 8 hpi, ∼95% of cells remained viable and metabolically active across all conditions. By 24 hpi, DFMO-treated cells retained ∼80% of metabolic activity and ∼90% of membrane integrity, while in all other PA-competent conditions, metabolic activity declined to less than 50% and viability to less than 25%. In PA-depleted spermine-supplemented cells, metabolic activity and membrane integrity showed an intermediate phenotype, similarly to what was observed above for the number of infected cells.

In summary, these findings demonstrate that PA depletion prevents loss of plasma membrane integrity, CPE, and cell death in cells that have been infected with a very high MOI of MNV-1 for 24 h, while still releasing 10^6^ TCID_50_/mL infectious units. Furthermore, exogenous PA reversed this phenotype, completely restoring the visible CPE.

### Restriction of MNV infection by PA depletion can be overcome by PI3K inhibition

Under PA-depleted conditions, inhibition of extrinsic apoptosis and cell death is attributed to sustained Akt kinase activation. Notably, modulation of the PI3K/Akt pathway by the reversible PI3K inhibitor LY294002 (LY) has been shown to restore homeostatic Akt levels under PA-depletion and to re-establish TNF-α-induced apoptosis in DFMO-treated intestinal epithelial cells (46).

**Fig 6:**
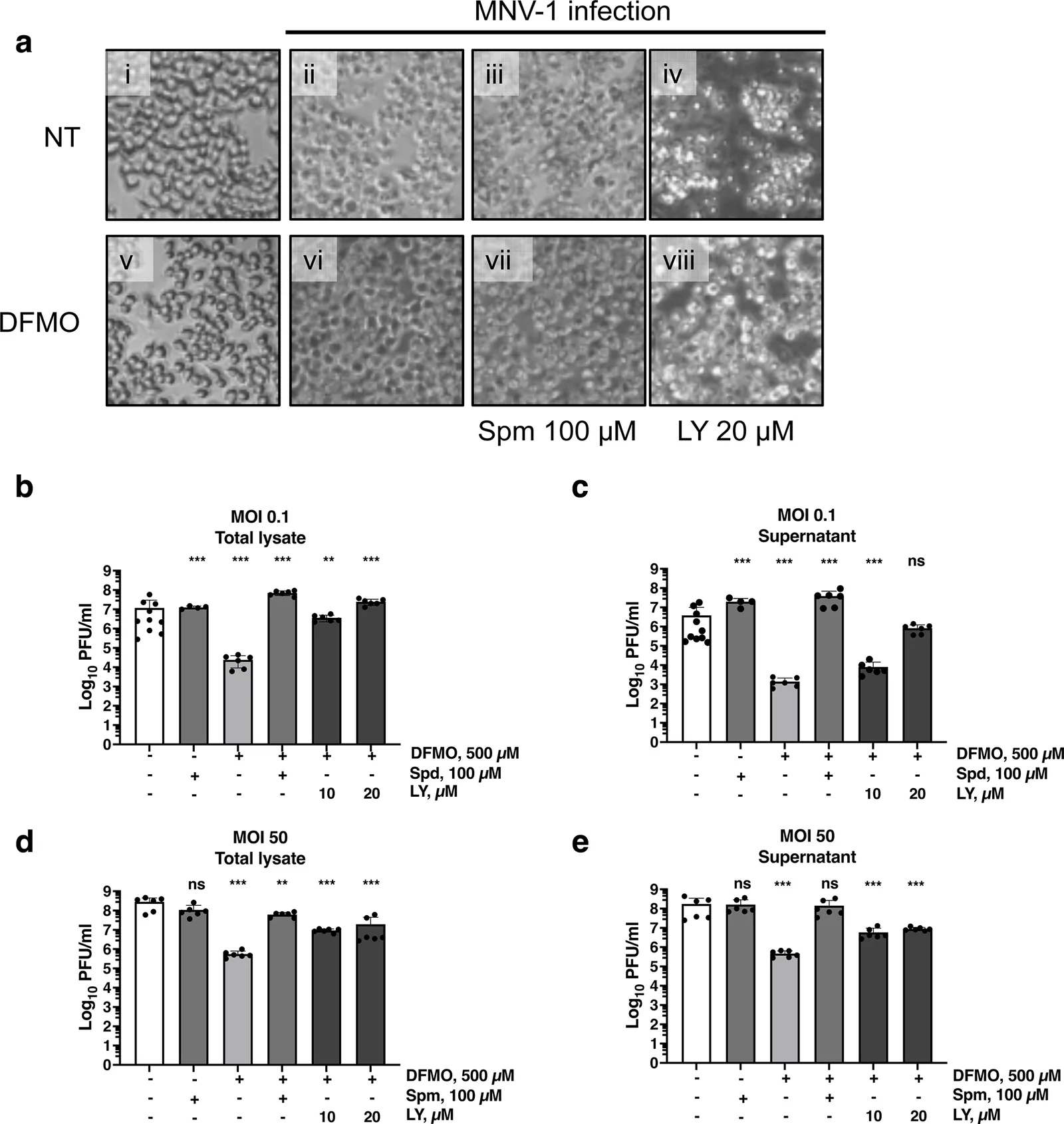
PI3K inhibition rescues MNV-1 infection and release in PA-depleted cells. RAW264.7 cells were pretreated with 500 μM DFMO for 4 days to deplete PAs and infected with MNV-1 at an MOI of 0.1 (**b and c**) or 50 (**a, d, and e**). Infected cells were supplemented with/without DFMO and/or 100 μM spermine (Spm), or 10–20 μM LY294002 (LY). MNV-induced CPE was analyzed 48 hpi by bright-field microscopy (**a**). Total lysates (**b and d**) or clarified supernatant (**c and e**) were harvested 48 hpi, and virus titers were determined by plaque assay. Data are presented as means ± standard deviation (SD) from three independent biological replicates with two technical replicates. Individual data points are shown. Statistical significance was assessed using the one-way ANOVA and shown as compared to non-treated: ns, not significant; **P ≤ 0.01; and ***P ≤ 0.001.

We therefore investigated whether PI3K inhibition could rescue MNV-1 infection in PA-depleted macrophages using the reversible PI3K inhibitor LY (Fig. 6). Treatment of PA-depleted cells (Fig. 6a, panel vi) with LY led to rescue of virus-induced CPE (Fig. 6a, panel viii). Infectious MNV-1 titers were determined 48 hpi at low (0.1) and high (50) MOI in total cell lysates (Fig. 6b and d) and supernatants (Fig. 6c and e) in the presence of either spermine or spermidine, or LY. Under low MOI infection, LY supplementation resulted in a dose-dependent rescue of MNV-1 replication and release from PA-depleted macrophages, comparable to spermidine supplementation. Notably, in total lysates, but not in supernatants, both LY and spermidine supplementation rescued MNV titers to levels observed in non-treated macrophages. Under high-MOI infection, LY partially rescued MNV-1 infection and release, with no difference observed between 10 and 20 µM LY treatment. In summary, we observed a significant rescue of MNV-1 infection and virus-induced CPE in PA-depleted macrophages under PI3K inhibition.

**Fig 7:**
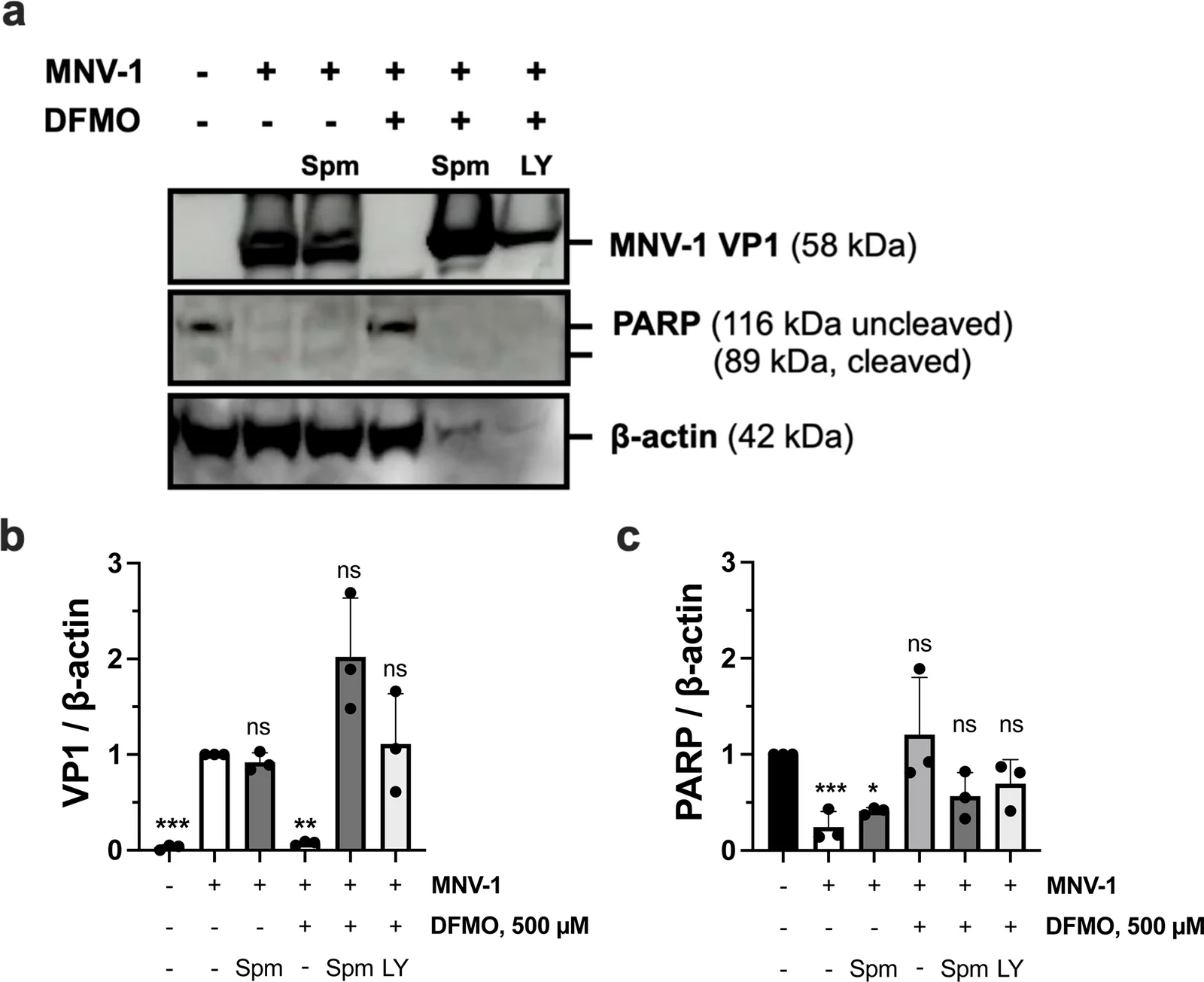
PI3K inhibition rescues MNV-1 capsid protein synthesis in PA-depleted cells. RAW264.7 cells were pretreated with 500 μM DFMO treatment for 4 days. Cells were then infected with MNV-1 at an MOI of 1 on ice for 1 h. Following infection, the inoculum was removed, and the medium was supplemented with/without DFMO, 10 μM LY294002 (LY), or 100 μM spermine (Spm). At 24 hpi, cell fractions were harvested for protein extraction and subsequently analyzed by western blotting to determine expression levels of MNV capsid protein VP1, PARP, and β-actin (β-act). A representative western blot is shown (**a**). A quantification of VP1 (**b**) and PARP (**c**) was performed using ImageJ gel analysis and normalized to β-actin. Data represent the means ± standard deviation (SD) of three independent biological assays (**b and c**). Individual data points are shown. For statistical analysis, PARP and VP1 expression were compared to the infected control using the one-way ANOVA test: ns, not significant (omitted for clarity); *P ≤ 0.05; **P ≤ 0.01; and ***P ≤ 0.001.

We next hypothesized that PI3K inhibition would also restore the virus-induced programmed cell death and the accumulation of MNV capsid protein in PA-depleted macrophages. To test this, we assessed PARP cleavage during MNV infection (Fig. 7), as caspase-3 activation and PARP cleavage have recently been identified as markers of MNV-induced apoptosis (25). Similarly to the reported observations in immortalized bone marrow-derived macrophages (26), our data confirmed that MNV-1 infection led to a loss of uncleaved PARP in PA-competent RAW264.7 cells. Interestingly, in DFMO-treated and infected cells, no virus-induced loss of PARP occurred 24 hpi. In infected PA-depleted RAW264.7 cells, spermine and LY restored PARP cleavage and MNV-1 VP1 accumulation. Under these conditions, a loss of β-actin was observed, potentially reflecting the increased cell death. No PARP cleavage occurred in uninfected LY and spermine-treated cells (Fig. S6), showing that PARP cleavage is a consequence of the infection and not of PI3K inhibition or PA reconstitution. In summary, CPE and loss of uncleaved PARP, indicative of apoptosis, correlated with the restoration of MNV infection and viral release in PA-depleted macrophages, which could be achieved by PA supplementation or modulation of PI3K activity.

### Exogenous cholesterol rescues MNV-1 replication and CPE under PA depletion

PA and PI3K/Akt signaling pathway are well-known modulators of host lipid metabolism. Reduced membrane cholesterol levels following PA depletion have been shown to impair coxsackievirus attachment and subsequent infection (61).

**Fig 8:**
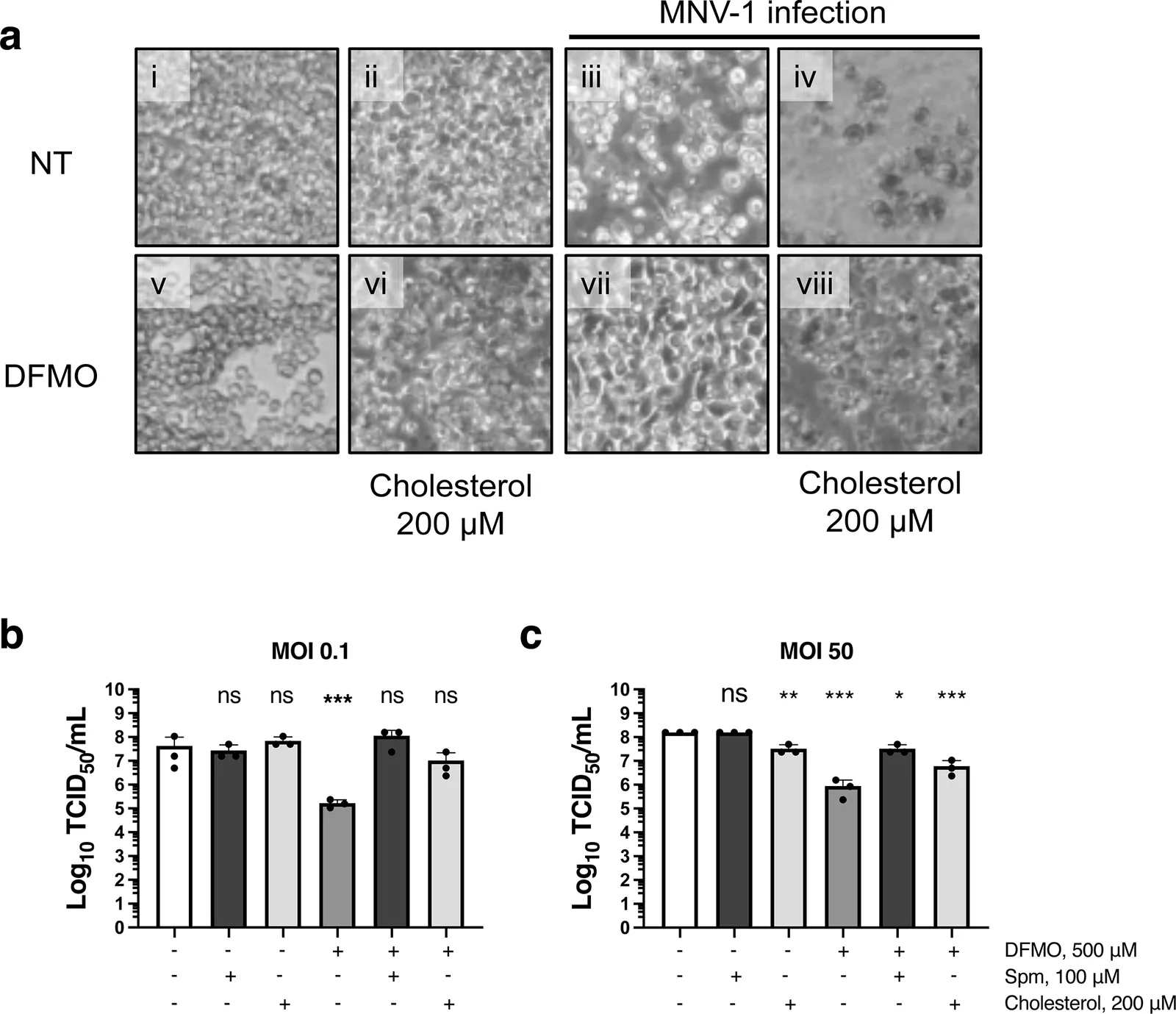
Exogenous cholesterol rescues MNV-1 replication and CPE under PA depletion. RAW264.7 cells were pretreated with 500 μM DFMO for 4 days to deplete PAs and infected with MNV-1 at an MOI of 0.1 (**b**) or 50 (**a and c**). Infected cells were supplemented with/without DFMO and/or 100 μM spermine (Spm), or 200 µM cholesterol. MNV-induced CPE was analyzed 48 hpi by bright-field microscopy (**a**). Total lysates (**b and c**) were harvested 48 hpi, and virus titers were determined by TCID_50_. Data are presented as means ± standard deviation (SD) from three independent biological repeats with two technical repeats. Individual data points are shown. Statistical significance was assessed using the one-way ANOVA and shown as compared to non-treated: ns, not significant; *P ≤ 0.05; **P ≤ 0.01; and ***P ≤ 0.001.

Although the initial steps of MNV infection, including attachment and entry, appear to be unaffected by PA depletion, as supported by the attachment assay (Fig. 2), MNV genome replication (Fig. 3), and the presence of dsRNA signals in infected cells (Fig. 5), we sought to determine whether exogenous cholesterol supplementation of PA-depleted macrophages could rescue MNV-1 infection at a step downstream of genome replication (Fig. 8). Here, we pretreated RAW264.7 cells for 4 days with DFMO and infected cells with MNV-1 at an MOI of 0.1 or 50. At 48 hpi, MNV-induced CPE was analyzed by light microscopy (Fig. 8a), and lysates were titered by endpoint titration (Fig. 8b and c). Upon cholesterol supplementation of PA-depleted cells, a rescue of virus-induced CPE in infected PA-depleted macrophages was observed (Fig. 8a, panels vii and viii). Under both low- and high-MOI infections, cholesterol supplementation partially rescued MNV-1 replication (Fig. 8b and c), supporting the hypothesis that PA-dependent regulation of host cholesterol homeostasis contributes to the establishment of productive MNV infection.

### PA depletion blocks productive HNoV GII.4 infection in HIEs

**Fig 9:**
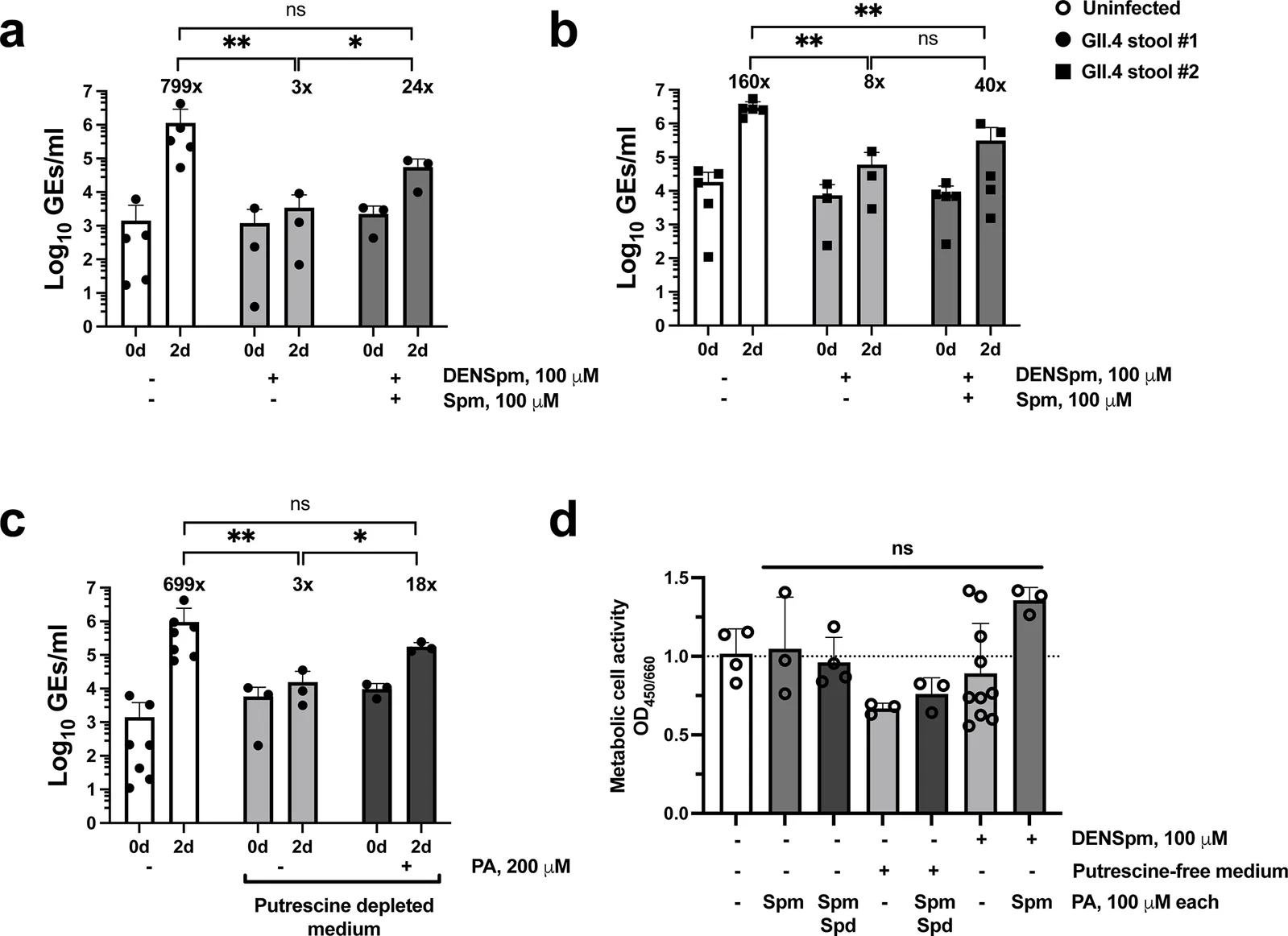
PA degradation inhibits HNoV GII.4 infection in HIEs. HIEs were differentiated in standard differentiation medium with 100 µM DENSpm, additionally added 2 days before infection (**a and b**), or 7 days in putrescine-free medium (B27^−^/N2^−^) (**c**). HIEs were infected with HNoV GII.4 stool isolates #1 (**a and c**) or #2 (**b**), at an approx. MOI of 1 for 1 h at 37°C. The inoculum was removed and replaced with standard medium with or without DENSpm and 100 µM Spm (**a and b**) or putrescine-free (B27^−^/N2^−^) medium with or without 200 µM PA mix (PA; 100 µM Spm + 100 µM Spd) (**c**). HNoV GEs were determined by RT-qPCR at 48 hpi. Numbers above the bars indicate the fold-change in viral GEs at 2 days compared to 0 days. Viability of uninfected, treated HIEs was assessed using the WST-1 metabolic assay (**d**). HIEs were treated as above, omitting only the viral inoculation. Data are presented as means ± standard deviation (SD) from a minimum of three independent biological assays. Individual data points are shown (**a–d**). Statistical significance was assessed using ANOVA in comparison to untreated/infected (**a–c**) or just untreated (**d**): ns, not significant; *P ≤ 0.05; and **P ≤ 0.01.

To assess whether PAs play a broad role in norovirus infection, we investigated the impact of PA depletion using two distinct HNoV stool isolates obtained from Lübeck University Clinic of Schleswig-Holstein (UKSH), genotyped as GII.4 Den Haag-like (Fig. 9). To assess the impact of PA restriction on HNoV infection, DENSpm was used to degrade PAs in HIEs, which resulted in full block of HNoV replication with partial titer rescue after supplementation with 100 µM spermine for both stool isolates (Fig. 9a and b). Since the HIE differentiation medium contains 150 µM putrescine, the use of DFMO was not feasible, as putrescine supports downstream PA synthesis. We therefore used a differentiation medium omitting the putrescine-containing additives B27 and N2 (putrescine-free medium [B27^−^/N2^−^]) (Fig. 9c). After 7 days of differentiation, HIEs were infected with both HNoV stool isolates at approximately MOI 1 (based on GEs). After inoculation, putrescine-free medium was reconstituted with a mix of 100 µM spermine and 100 µM spermidine. HNoV titers were quantified 48 hpi from whole cell samples via RT-qPCR. Depletion of putrescine from differentiation medium resulted in full restriction of HNoV infection, and supplementing PAs resulted in a partial rescue. Cell viability of HIEs was not significantly affected in PA-depleted versus reconstituted conditions (Fig. 9d). Differentiation in putrescine-depleted medium led to ~30% loss in viability; this was, however, not significantly different from the reconstituted condition. An increase in metabolic activity was observed in DENSpm-treated spermine-supplemented HIEs, similarly to what was observed for murine cells (Fig. S3). Collectively, our data demonstrate that PA availability is a critical determinant of productive HNoV infection in human intestinal enteroids, with PA depletion strongly restricting viral replication independently of the viral isolate or PA depletion strategy used.

## DISCUSSION

Viruses have evolved diverse strategies to manipulate host cellular pathways to acquire essential biomolecules and establish an intracellular environment conducive to infection. PAs are ubiquitous cellular metabolites present at high micromolar concentrations that regulate a broad range of cellular processes (31–35). Consequently, PAs have emerged as important host factors supporting the replication of both RNA and DNA viruses (28). Several RNA viruses, including enteric viruses such as poliovirus, coxsackievirus B3 (CVB3), and enterovirus A71, depend on host-derived PAs for efficient replication (29, 53). Our study extends this dependency to noroviruses. We demonstrate that PA restriction in naturally susceptible murine immune cells and recombinant human epithelial cells significantly impairs MNV infection, whereas PA supplementation restores viral replication. Similarly, PA depletion in HIEs strongly inhibits HNoV GII.4 replication, indicating that noroviruses exploit host PA availability to support productive infection, consistent with observations for flaviviruses, coronaviruses, phleboviruses, and enteroviruses (53).

Prior studies have linked PAs to virus attachment and replication of enteroviruses (61) and flaviviruses (53). PAs were shown to affect the activity of transcription factor SREBP2, a crucial factor for cholesterol biosynthesis. Notably, cholesterol supplementation in PA-depleted cells rescued enterovirus CVB3 infection (36). Although MNV was shown to require cholesterol for cell entry (57), we found that PA depletion did not impact MNV attachment or entry to susceptible cells.

Many flaviviruses rely on host-derived PAs for viral genome replication and protein translation; we, on the other hand, observed no reduction in MNV genome replication in PA-depleted murine macrophages (Fig. 3). This was similar to hepatitis B virus, where it was shown that DFMO inhibits HBV replication by reducing HBc protein stability without affecting mRNA transcription or protein translation (62). In the case of MNV, PA depletion significantly diminished major capsid protein accumulation in infected macrophages (Fig. 8).

Our findings indicate that noroviruses rely on host-derived PAs to support capsid protein accumulation and productive infection. PA depletion markedly reduced MNV capsid protein accumulation, prevented the expansion of virus-positive cells beyond the initial round of infection, and diminished virus-induced cell death. In PA-depleted macrophages, reduced CPE was associated with preserved cellular viability and metabolic activity, maintained membrane integrity, and decreased PARP cleavage. Supplementation with spermine restored MNV titers, capsid protein accumulation, and apoptotic markers, highlighting the critical role of PAs in MNV infection. Furthermore, modulation of PI3K activity with LY or supplementation with exogenous cholesterol restored MNV titers and virus-induced cell death in PA-depleted macrophages, suggesting that PA-dependent regulation of host signaling and lipid homeostasis contributes to productive infection.

PAs have been implicated in the regulation of intracellular cholesterol homeostasis and trafficking. Spermidine supplementation reduces plasma membrane cholesterol levels (63) and promotes cholesterol efflux in a cell type-dependent manner (64). In contrast, disruption of lysosomal PA homeostasis, either through inactivation of the endosomal PA transporter ATP13A2 (65) or supplementation with U18666A {3β-[2-(diethylamino)ethoxy]androst-5-en-17-one}, a cell-permeable amphipathic tertiary amine, inhibits cholesterol trafficking from lysosomes to the plasma membrane and Golgi apparatus (65, 66). Notably, U18666A also potently inhibits PA-dependent viruses, including CVB3 and bunyaviruses (36, 42). Although DFMO treatment has been shown to broadly reduce cholesterol synthesis (36), staining for polar and neutral lipids revealed altered lipid distribution and accumulation in PA-depleted cells (67). Therefore, it will be important to determine whether PA depletion affects compartment-specific cholesterol trafficking and redistribution, and how these alterations influence MNV infection.

Overall, these observations suggest that PI3K signaling and downstream targets such as cholesterol homeostasis (68–70) contribute to the regulation of MNV infection and virus-induced cell death. Together, these findings indicate that host PAs influence multiple stages of MNV infection in murine macrophages, including capsid protein accumulation, productive replication, and the induction of virus-associated cell death.

While the PI3K/Akt molecular pathway is involved in many cellular processes (71), it is well established that activation of Akt protein via phosphorylation by PI3K prevents apoptosis and prolongs cell survival (72, 73). MNV induces Akt phosphorylation at early time points of infection to support cell survival during virus replication. Inhibiting Akt phosphorylation has been shown to interfere with the optimum assembly of MNV virions and to promote early virus release, in a strain-dependent manner (20). Thus, precise Akt protein regulation appears critical for MNV egress. Restricting PAs is reported to lead to sustained Akt phosphorylation (38, 74, 75), which might alter infection dynamics and reduce apoptosis by inhibiting key events like PARP cleavage and phosphatidylserine availability (76). Assessing the abundance of total Akt and phosphorylated Akt protein together with cleaved PARP protein and intracellular levels of cholesterol with inhibition of PI3K under PA depletion would further clarify the role of PA regulation of the PI3K/Akt/cholesterol axis in norovirus-induced apoptosis.

One potential mechanism involves hypusination, a spermidine-dependent post-translational modification of eIF5A that regulates translation elongation, particularly for proteins containing polyproline sequences or other ribosome-stalling motifs that are otherwise challenging to synthesize efficiently. Although the norovirus genome does not contain obvious polyproline stretches, hypusination may influence norovirus replication through alternative mechanisms, including regulation of the ribosomal frameshifting event required for ORF3 translation (77).

Our study also has limitations that provide avenues for future investigation. While we assessed PA restriction of MNV infection *in vitro* across several different cell types, extending these studies to primary murine macrophages and dendritic cells would further validate the role of PAs in MNV infection in physiologically relevant systems. Importantly, the ability of PA restriction to inhibit HNoV infection in the HIE model already supports the biological relevance of these findings in a non-transformed human system. Although we excluded effects on early stages of infection, including viral attachment and genome replication, and narrowed the critical step to a stage between genome replication and viral release, the underlying mechanism remains to be defined. Future studies should focus on the potential roles of PAs in viral protein translation and virion assembly.

Another open question is the impact *in vivo*. PA depletion significantly attenuated CVB3 and CHIKV replication in multiple organs of infected mice (53). PA supplementation in mice also enhanced herpes simplex virus 1 (HSV-1) infection (77). Mice treated with DENSP showed markedly reduced HSV-1 infections in the brain and spleen. PAs also facilitate alphavirus infection *in vivo*, as shown for Sindbis virus (SINV) infection in the *Drosophila melanogaster* and zebrafish (*Danio rerio*) model. Here, animals were fed DFMO for 7 days prior to infection, resulting in a significant reduction in infection (59). These data show that PA modulation *in vivo* by supplementation or pharmacological depletion is an effective strategy against a broad range of PA-dependent viruses.

Pancreatic ductal cells persistently infected with CVB3 lacked a robust virus-induced CPE and showed reduced PA levels (56). A similar situation might be possible for persistent MNV strains, such as CR3, which reside in an epithelial reservoir, by downregulating IFN-λ signaling (19, 78). A direct feedback loop in which IFNs induce SAT1 expression, leading to PA depletion in a manner similar to DENSp, has also been described for Zika and CHIKV, leading to restricted infection (59). Whether PA depletion occurs in MNV-infected tuft cells or contributes to MNV persistence is an intriguing question that warrants further study.

In addition to intrinsic PA, gut bacteria are also a major source of PA in the intestine (33, 79, 80). The absence of commensal bacteria, that is, by antibiotic treatment, would directly affect PA levels in the gut. Antibiotic treatment has been linked with reduced MNV infection in the ileum and colon and protection from disease (78, 81, 82), adding an extra layer of complexity that needs to be considered for *in vivo* analysis and therapeutic approaches.

Understanding the role of host PAs in norovirus infection could guide antiviral therapy development. The PA synthesis inhibitor DFMO is currently in clinical use for trypanosomiasis (83) and demonstrates good tolerability (84). The PA analog DENSpm also has a good safety profile (85–87), although it still lacks clinical approval. Significant PA depletion requires 24–96 h *in vitro*. While this indicates that PA inhibition may be less effective against acute norovirus infection, PA restriction may offer a promising therapeutic approach for patients with chronic norovirus infection, who suffer from prolonged diarrhea and are unable to clear the infection. However, any therapeutic application would require considerations regarding external sources of PA in the gut, taking dietary PA uptake (88) and PA production by gut commensals (80) into account.

### Summary

In summary, our research highlights the role of PAs in norovirus infection. We demonstrate that restricting PAs reversibly inhibits MNV and HNoV infection. For MNV, we were able to show that PA depletion disrupts capsid protein production and subsequent reinfection of the cells. To the best of our knowledge, this is the first report demonstrating that MNV requires PAs. A role for cholesterol was identified during a late stage of the infectious cycle, involving capsid protein synthesis. Our findings identify a novel target to exploit for intervention against norovirus infection, open to already licensed pharmacological approaches such as DFMO, as well as dietary and targeted microbiome modulation approaches to deplete PAs in the gut.

## SUPPLEMENTAL MATERIAL

Supplemental Figures mbio.02033-26-s0001.pdf Figures S1 to S6.

ASM does not own the copyrights to Supplemental Material that may be linked to, or accessed through, an article. The authors have granted ASM a non-exclusive, world-wide license to publish the Supplemental Material files. Please contact the corresponding author directly for reuse.

## References

[1] Wobus CE, Green KY. 2020. Edited by P. M. Howley and D. M. Knipe. Fields virology: emerging viruses. 7th ed. Vol. 1. Lippincott Williams & Wilkins.

[2] Glass RI, Parashar UD, Estes MK. 2009. Norovirus gastroenteritis. N Engl J Med 361:1776–1785. doi:10.1056/NEJMra0804575 PMC3880795 19864676

[3] Bok K, Green KY. 2012. Norovirus gastroenteritis in immunocompromised patients. N Engl J Med 367:2126–2132. doi:10.1056/NEJMra1207742 PMC4944753 23190223

[4] Prasad BVV, Atmar RL, Ramani S, Palzkill T, Song Y, Crawford SE, Estes MK. 2025. Norovirus replication, host interactions and vaccine advances. Nat Rev Microbiol 23:385–401. doi:10.1038/s41579-024-01144-9 PMC12088900 39824927

[5] Cheng C, Cai X, Li J, Zhang X, Xie Y, Zhang J. 2024. *In vitro* culture of human norovirus in the last 20 years. Biomedicines 12:2442. doi:10.3390/biomedicines12112442 PMC11592199 39595008

[6] Graziano VR, Walker FC, Kennedy EA, Wei J, Ettayebi K, Strine MS, Filler RB, Hassan E, Hsieh LL, Kim AS, Kolawole AO, Wobus CE, Lindesmith LC, Baric RS, Estes MK, Orchard RC, Baldridge MT, Wilen CB. 2020. CD300lf is the primary physiologic receptor of murine norovirus but not human norovirus. PLoS Pathog 16:e1008242. doi:10.1371/journal.ppat.1008242 PMC7162533 32251490

[7] Ettayebi K, Crawford SE, Murakami K, Broughman JR, Karandikar U, Tenge VR, Neill FH, Blutt SE, Zeng X-L, Qu L, Kou B, Opekun AR, Burrin D, Graham DY, Ramani S, Atmar RL, Estes MK. 2016. Replication of human noroviruses in stem cell-derived human enteroids. Science 353:1387–1393. doi:10.1126/science.aaf5211 PMC5305121 27562956

[8] Ettayebi K, Tenge VR, Cortes-Penfield NW, Crawford SE, Neill FH, Zeng XL, Yu X, Ayyar BV, Burrin D, Ramani S, Atmar RL, Estes MK. 2021. New insights and enhanced human norovirus cultivation in human intestinal enteroids. mSphere 6:e01136-20. doi:10.1128/mSphere.01136-20 PMC7885322 33504663

[9] Lewis MA, Cortés-Penfield NW, Ettayebi K, Patil K, Kaur G, Neill FH, Atmar RL, Ramani S, Estes MK. 2023. Standardization of an antiviral pipeline for human norovirus in human intestinal enteroids demonstrates nitazoxanide has no to weak antiviral activity. Antimicrob Agents Chemother 67:e00636-23. doi:10.1128/aac.00636-23 PMC10583671 37787556

[10] Karst SM, Wobus CE, Lay M, Davidson J, Virgin HW 4th. 2003. STAT1-dependent innate immunity to a Norwalk-like virus. Science 299:1575–1578. doi:10.1126/science.1077905 12624267

[11] Thackray LB, Wobus CE, Chachu KA, Liu B, Alegre ER, Henderson KS, Kelley ST, Virgin HW 4th. 2007. Murine noroviruses comprising a single genogroup exhibit biological diversity despite limited sequence divergence. J Virol 81:10460–10473. doi:10.1128/JVI.00783-07 PMC2045448 17652401

[12] Wobus CE, Thackray LB, Virgin HW 4th. 2006. Murine norovirus: a model system to study norovirus biology and pathogenesis. J Virol 80:5104–5112. doi:10.1128/JVI.02346-05 PMC1472167 16698991

[13] Orchard RC, Wilen CB, Doench JG, Baldridge MT, McCune BT, Lee Y-CJ, Lee S, Pruett-Miller SM, Nelson CA, Fremont DH, Virgin HW. 2016. Discovery of a proteinaceous cellular receptor for a norovirus. Science 353:933–936. doi:10.1126/science.aaf1220 PMC5484048 27540007

[14] Haga K, Fujimoto A, Takai-Todaka R, Miki M, Doan YH, Murakami K, Yokoyama M, Murata K, Nakanishi A, Katayama K. 2016. Functional receptor molecules CD300lf and CD300ld within the CD300 family enable murine noroviruses to infect cells. Proc Natl Acad Sci USA 113:E6248–E6255. doi:10.1073/pnas.1605575113 PMC5068309 27681626

[15] Cox C, Cao S, Lu Y. 2009. Enhanced detection and study of murine norovirus-1 using a more efficient microglial cell line. Virol J 6:196. doi:10.1186/1743-422X-6-196 PMC2777878 19903359

[16] Wobus CE, Karst SM, Thackray LB, Chang KO, Sosnovtsev SV, Belliot G, Krug A, Mackenzie JM, Green KY, Virgin HW. 2004. Replication of *Norovirus* in cell culture reveals a tropism for dendritic cells and macrophages. PLoS Biol 2:e432. doi:10.1371/journal.pbio.0020432 PMC532393 15562321

[17] Lee S, Wilen CB, Orvedahl A, McCune BT, Kim KW, Orchard RC, Peterson ST, Nice TJ, Baldridge MT, Virgin HW. 2017. Norovirus cell tropism is determined by combinatorial action of a viral non-structural protein and host cytokine. Cell Host Microbe 22:449–459. doi:10.1016/j.chom.2017.08.021 PMC5679710 28966054

[18] Wilen CB, Lee S, Hsieh LL, Orchard RC, Desai C, Hykes BL, McAllaster MR, Balce DR, Feehley T, Brestoff JR, Hickey CA, Yokoyama CC, Wang Y-T, MacDuff DA, Kreamalmayer D, Howitt MR, Neil JA, Cadwell K, Allen PM, Handley SA, van Lookeren Campagne M, Baldridge MT, Virgin HW. 2018. Tropism for tuft cells determines immune promotion of norovirus pathogenesis. Science 360:204–208. doi:10.1126/science.aar3799 PMC6039974 29650672

[19] Strine MS, Fagerberg E, Darcy PW, Barrón GM, Filler RB, Alfajaro MM, D’Angelo-Gavrish N, Wang F, Graziano VR, Menasché BL, Damo M, Wang Y-T, Howitt MR, Lee S, Joshi NS, Mucida D, Wilen CB. 2024. Intestinal tuft cell immune privilege enables norovirus persistence. Sci Immunol 9:eadi7038. doi:10.1126/sciimmunol.adi7038 PMC11555782 38517952

[20] Owusu IA, Passalacqua KD, Mirabelli C, Lu J, Young VL, Hosmillo M, Quaye O, Goodfellow I, Ward VK, Wobus CE. 2022. Akt plays differential roles during the life cycles of acute and persistent murine norovirus strains in macrophages. J Virol 96:e01923-21. doi:10.1128/JVI.01923-21 PMC8827033 34787460

[21] Jones MK, Watanabe M, Zhu S, Graves CL, Keyes LR, Grau KR, Gonzalez-Hernandez MB, Iovine NM, Wobus CE, Vinjé J, Tibbetts SA, Wallet SM, Karst SM. 2014. Enteric bacteria promote human and mouse norovirus infection of B cells. Science 346:755–759. doi:10.1126/science.1257147 PMC4401463 25378626

[22] Blanco J, Cameirao C, López MC, Muñoz-Barroso I. 2020. Phosphatidylinositol-3-kinase-Akt pathway in negative-stranded RNA virus infection: a minireview. Arch Virol 165:2165–2176. doi:10.1007/s00705-020-04740-1 32740830

[23] Wang G, Zhang D, Orchard RC, Hancks DC, Reese TA. 2023. Norovirus MLKL-like protein initiates cell death to induce viral egress. Nature 616:152–158. doi:10.1038/s41586-023-05851-w PMC10348409 36991121

[24] Aktepe TE, Deerain JM, Hyde JL, Fritzlar S, Mead EM, Carrera Montoya J, Hachani A, Pearson JS, White PA, Mackenzie JM. 2024. Norovirus-mediated translation repression promotes macrophage cell death. PLoS Pathog 20:e1012480. doi:10.1371/journal.ppat.1012480 PMC11398682 39226332

[25] Yen JB, Chen LW, Wei LH, Hung CH, Wang SS, Lin CL, Chang PJ. 2021. Identification and characterization of human norovirus NTPase regions required for lipid droplet localization, cellular apoptosis, and interaction with the viral P22 protein. Microbiol Spectr 9:e00422-21. doi:10.1128/spectrum.00422-21 PMC8552650 34431704

[26] Deerain JM, Aktepe TE, Trenerry AM, Ebert G, Hyde JL, Charry K, Edgington-Mitchell L, Xu B, Ambrose RL, Sarvestani ST, Lawlor KE, Pearson JS, White PA, Mackenzie JM. 2024. Murine norovirus infection of macrophages induces intrinsic apoptosis as the major form of programmed cell death. Virology 589:109921. doi:10.1016/j.virol.2023.109921 37939648

[27] Yen JB, Wei LH, Chen LW, Chen LY, Hung CH, Wang SS, Chang PJ. 2018. Subcellular localization and functional characterization of GII.4 norovirus-encoded NTPase. J Virol 92:e01824-17. doi:10.1128/JVI.01824-17 PMC5809722 29212938

[28] Firpo MR, Mounce BC. 2020. Diverse functions of polyamines in virus infection. Biomolecules 10:628. doi:10.3390/biom10040628 PMC7226272 32325677

[29] Mounce BC, Olsen ME, Vignuzzi M, Connor JH. 2017. Polyamines and their role in virus infection. Microbiol Mol Biol Rev 81:e00029-17. doi:10.1128/MMBR.00029-17 PMC5706744 28904024

[30] Nakamura A, Matsumoto M. 2025. Role of polyamines in intestinal mucosal barrier function. Semin Immunopathol 47:9. doi:10.1007/s00281-024-01035-4 PMC11750915 39836273

[31] Igarashi K, Kashiwagi K. 2010. Modulation of cellular function by polyamines. Int J Biochem Cell Biol 42:39–51. doi:10.1016/j.biocel.2009.07.009 19643201

[32] Pegg AE. 2016. Functions of polyamines in mammals. J Biol Chem 291:14904–14912. doi:10.1074/jbc.R116.731661 PMC4946908 27268251

[33] Pegg AE. 2018. Introduction to the Thematic Minireview Series: sixty plus years of polyamine research. J Biol Chem 293:18681–18692. doi:10.1074/jbc.TM118.006291 PMC6290159 30377254

[34] Madeo F, Eisenberg T, Pietrocola F, Kroemer G. 2018. Spermidine in health and disease. Science 359:eaan2788. doi:10.1126/science.aan2788 29371440

[35] Sánchez-Jiménez F, Medina MÁ, Villalobos-Rueda L, Urdiales JL. 2019. Polyamines in mammalian pathophysiology. Cell Mol Life Sci 76:3987–4008. doi:10.1007/s00018-019-03196-0 PMC11105599 31227845

[36] Firpo MR, LoMascolo NJ, Petit MJ, Shah PS, Mounce BC. 2023. Polyamines and eIF5A hypusination facilitate SREBP2 synthesis and cholesterol production leading to enhanced enterovirus attachment and infection. PLoS Pathog 19:e1011317. doi:10.1371/journal.ppat.1011317 PMC10146511 37071661

[37] Nitta T, Igarashi K, Yamamoto N. 2002. Polyamine depletion induces apoptosis through mitochondria-mediated pathway. Exp Cell Res 276:120–128. doi:10.1006/excr.2002.5517 11978014

[38] Zhang HM, Rao JN, Guo X, Liu L, Zou T, Turner DJ, Wang JY. 2004. Akt kinase activation blocks apoptosis in intestinal epithelial cells by inhibiting caspase-3 after polyamine depletion. J Biol Chem 279:22539–22547. doi:10.1074/jbc.M314337200 15024023

[39] Routhu NK, Xie Y, Dunworth M, Casero RA, Oupicky D, Byrareddy SN. 2018. Polymeric prodrugs targeting polyamine metabolism inhibit Zika virus replication. Mol Pharm 15:4284–4295. doi:10.1021/acs.molpharmaceut.8b00068 PMC6538062 30040423

[40] Cruz-Pulido YE, Mounce BC. 2023. Good cop, bad cop: polyamines play both sides in host immunity and viral replication. Semin Cell Dev Biol 146:70–79. doi:10.1016/j.semcdb.2022.12.004 PMC10101871 36604249

[41] Mastrodomenico V, Esin JJ, Graham ML, Tate PM, Hawkins GM, Sandler ZJ, Rademacher DJ, Kicmal TM, Dial CN, Mounce BC. 2019. Polyamine depletion inhibits bunyavirus infection via generation of noninfectious interfering virions. J Virol 93:e00530-19. doi:10.1128/JVI.00530-19 PMC6600196 31043534

[42] Mastrodomenico V, LoMascolo NJ, Cruz-Pulido YE, Cunha CR, Mounce BC. 2022. Polyamine-linked cholesterol incorporation in Rift Valley fever virus particles promotes infectivity. ACS Infect Dis 8:1439–1448. doi:10.1021/acsinfecdis.2c00071 PMC9549488 35786847

[43] Wobus CE, Peiper AM, McSweeney AM, Young VL, Chaika M, Lane MS, Lingemann M, Deerain JM, Strine MS, Alfajaro MM, Helm EW, Karst SM, Mackenzie JM, Taube S, Ward VK, Wilen CB. 2023. Murine norovirus: additional protocols for basic and antiviral studies. Curr Protoc 3:e828. doi:10.1002/cpz1.828 PMC10375541 37478303

[44] Creutznacher R, Maass T, Dülfer J, Feldmann C, Hartmann V, Lane MS, Knickmann J, Westermann LT, Thiede L, Smith TJ, Uetrecht C, Mallagaray A, Waudby CA, Taube S, Peters T. 2022. Distinct dissociation rates of murine and human norovirus P-domain dimers suggest a role of dimer stability in virus-host interactions. Commun Biol 5:563. doi:10.1038/s42003-022-03497-4 PMC9184547 35680964

[45] Hwang S, Alhatlani B, Arias A, Caddy SL, Christodoulou C, Cunha JB, Emmott E, Gonzalez-Hernandez M, Kolawole A, Lu J, Rippinger C, Sorgeloos F, Thorne L, Vashist S, Goodfellow I, Wobus CE. 2014. Murine norovirus: propagation, quantification, and genetic manipulation. Curr Protoc Microbiol 33:15K. doi:10.1002/9780471729259.mc15k02s33 PMC4074558 24789596

[46] Reed LJ, Muench H. 1938. A simple method of estimating fifty per cent endpoints. Am J Epidemiol 27:493–497. doi:10.1093/oxfordjournals.aje.a118408

[47] Lei C, Yang J, Hu J, Sun X. 2021. On the calculation of TCID_50_ for quantitation of virus infectivity. Virol Sin 36:141–144. doi:10.1007/s12250-020-00230-5 PMC7973348 32458296

[48] Parra GI, Azure J, Fischer R, Bok K, Sandoval-Jaime C, Sosnovtsev SV, Sander P, Green KY. 2013. Identification of a broadly cross-reactive epitope in the inner shell of the norovirus capsid. PLoS One 8:e67592. doi:10.1371/journal.pone.0067592 PMC3689733 23805319

[49] Schägger H, von Jagow G. 1987. Tricine-sodium dodecyl sulfate-polyacrylamide gel electrophoresis for the separation of proteins in the range from 1 to 100 kDa. Anal Biochem 166:368–379. doi:10.1016/0003-2697(87)90587-2 2449095

[50] Niendorf S, Jacobsen S, Faber M, Eis-Hübinger AM, Hofmann J, Zimmermann O, Höhne M, Bock CT. 2017. Steep rise in norovirus cases and emergence of a new recombinant strain GII.P16-GII.2, Germany, winter 2016. Euro Surveill 22:30447. doi:10.2807/1560-7917.ES.2017.22.4.30447 PMC5388089 28181902

[51] Bernard H, Höhne M, Niendorf S, Altmann D, Stark K. 2014. Epidemiology of norovirus gastroenteritis in Germany 2001–2009: eight seasons of routine surveillance. Epidemiol Infect 142:63–74. doi:10.1017/S0950268813000435 PMC9152553 23517686

[52] Mirabelli C, Santos-Ferreira N, Gillilland MG 3rd, Cieza RJ, Colacino JA, Sexton JZ, Neyts J, Taube S, Rocha-Pereira J, Wobus CE. 2022. Human norovirus efficiently replicates in differentiated 3D-human intestinal enteroids. J Virol 96:e00855-22. doi:10.1128/jvi.00855-22 PMC9683019 36342297

[53] Mounce BC, Cesaro T, Moratorio G, Hooikaas PJ, Yakovleva A, Werneke SW, Smith EC, Poirier EZ, Simon-Loriere E, Prot M, Tamietti C, Vitry S, Volle R, Khou C, Frenkiel MP, Sakuntabhai A, Delpeyroux F, Pardigon N, Flamand M, Barba-Spaeth G, Lafon M, Denison MR, Albert ML, Vignuzzi M. 2016. Inhibition of polyamine biosynthesis is a broad-spectrum strategy against RNA viruses. J Virol 90:9683–9692. doi:10.1128/JVI.01347-16 PMC5068521 27535047

[54] Murray Stewart T, Dunston TT, Woster PM, Casero RA Jr. 2018. Polyamine catabolism and oxidative damage. J Biol Chem 293:18736–18745. doi:10.1074/jbc.TM118.003337 PMC6290137 30333229

[55] Kicmal TM, Tate PM, Dial CN, Esin JJ, Mounce BC. 2019. Polyamine depletion abrogates enterovirus cellular attachment. J Virol 93:e01054-19. doi:10.1128/JVI.01054-19 PMC6798109 31341056

[56] Mastrodomenico V, LoMascolo NJ, Firpo MR, Villanueva Guzman MDM, Zaporowski A, Mounce BC. 2023. Persistent coxsackievirus B3 infection in pancreatic ductal cells *in vitro* downregulates cellular polyamine metabolism. mSphere 8:e00036-23. doi:10.1128/msphere.00036-23 PMC10286709 37097178

[57] Perry JW, Wobus CE. 2010. Endocytosis of murine norovirus 1 into murine macrophages is dependent on dynamin II and cholesterol. J Virol 84:6163–6176. doi:10.1128/JVI.00331-10 PMC2876640 20375172

[58] Gerondopoulos A, Jackson T, Monaghan P, Doyle N, Roberts LO. 2010. Murine norovirus-1 cell entry is mediated through a non-clathrin-, non-caveolae-, dynamin- and cholesterol-dependent pathway. J Gen Virol 91:1428–1438. doi:10.1099/vir.0.016717-0 20147520

[59] Mounce BC, Poirier EZ, Passoni G, Simon-Loriere E, Cesaro T, Prot M, Stapleford KA, Moratorio G, Sakuntabhai A, Levraud JP, Vignuzzi M. 2016. Interferon-induced spermidine-spermine acetyltransferase and polyamine depletion restrict Zika and Chikungunya viruses. Cell Host Microbe 20:167–177. doi:10.1016/j.chom.2016.06.011 27427208

[60] Mounce BC, Cesaro T, Vlajnić L, Vidiņa A, Vallet T, Weger-Lucarelli J, Passoni G, Stapleford KA, Levraud J-P, Vignuzzi M. 2017. Chikungunya virus overcomes polyamine depletion by mutation of nsp1 and the opal stop codon to confer enhanced replication and fitness. J Virol 91:e00344-17. doi:10.1128/JVI.00344-17 PMC5512238 28539441

[61] Dial CN, Tate PM, Kicmal TM, Mounce BC. 2019. Coxsackievirus B3 responds to polyamine depletion via enhancement of 2A and 3C protease activity. Viruses 11:403. doi:10.3390/v11050403 PMC6563312 31052199

[62] Mao B, Wang Z, Pi S, Long Q, Chen K, Cui J, Huang A, Hu Y. 2020. Difluoromethylornithine, a decarboxylase 1 inhibitor, suppresses hepatitis B virus replication by reducing HBc protein levels. Front Cell Infect Microbiol 10:158. doi:10.3389/fcimb.2020.00158 PMC7176913 32373551

[63] Hibino S, Eto S, Hangai S, Endo K, Ashitani S, Sugaya M, Osawa T, Soga T, Taniguchi T, Yanai H. 2023. Tumor cell–derived spermidine is an oncometabolite that suppresses TCR clustering for intratumoral CD8^+^ T cell activation. Proc Natl Acad Sci USA 120:e2305245120. doi:10.1073/pnas.2305245120 PMC10268234 37276392

[64] Michiels CF, Kurdi A, Timmermans J-P, De Meyer GRY, Martinet W. 2016. Spermidine reduces lipid accumulation and necrotic core formation in atherosclerotic plaques via induction of autophagy. Atherosclerosis 251:319–327. doi:10.1016/j.atherosclerosis.2016.07.899 27450786

[65] Cenedella RJ. 2009. Cholesterol synthesis inhibitor U18666A and the role of sterol metabolism and trafficking in numerous pathophysiological processes. Lipids 44:477–487. doi:10.1007/s11745-009-3305-7 19440746

[66] Lu F, Liang Q, Abi-Mosleh L, Das A, De Brabander JK, Goldstein JL, Brown MS. 2015. Identification of NPC1 as the target of U18666A, an inhibitor of lysosomal cholesterol export and Ebola infection. eLife 4:e12177. doi:10.7554/eLife.12177 PMC4718804 26646182

[67] Cruz-Pulido YE, LoMascolo NJ, May D, Hatahet J, Thomas CE, Chu AKW, Stacey SP, Villanueva Guzman MDM, Aubert G, Mounce BC. 2024. Polyamines mediate cellular energetics and lipid metabolism through mitochondrial respiration to facilitate virus replication. PLoS Pathog 20:e1012711. doi:10.1371/journal.ppat.1012711 PMC11611256 39556649

[68] Kumar N, Mandal CC. 2021. Cholesterol-lowering drugs on Akt signaling for prevention of tumorigenesis. Front Genet 12:724149. doi:10.3389/fgene.2021.724149 PMC8483559 34603386

[69] Porstmann T, Santos CR, Griffiths B, Cully M, Wu M, Leevers S, Griffiths JR, Chung Y-L, Schulze A. 2008. SREBP activity is regulated by mTORC1 and contributes to Akt-dependent cell growth. Cell Metab 8:224–236. doi:10.1016/j.cmet.2008.07.007 PMC2593919 18762023

[70] Luu W, Sharpe LJ, Stevenson J, Brown AJ. 2012. Akt acutely activates the cholesterogenic transcription factor SREBP-2. Biochim Biophys Acta 1823:458–464. doi:10.1016/j.bbamcr.2011.09.017 22005015

[71] Manning BD, Toker A. 2017. AKT/PKB signaling: navigating the network. Cell 169:381–405. doi:10.1016/j.cell.2017.04.001 PMC5546324 28431241

[72] Zhou H, Li XM, Meinkoth J, Pittman RN. 2000. Akt regulates cell survival and apoptosis at a postmitochondrial level. J Cell Biol 151:483–494. doi:10.1083/jcb.151.3.483 PMC2185587 11062251

[73] Luo HR, Hattori H, Hossain MA, Hester L, Huang Y, Lee-Kwon W, Donowitz M, Nagata E, Snyder SH. 2003. Akt as a mediator of cell death. Proc Natl Acad Sci USA 100:11712–11717. doi:10.1073/pnas.1634990100 PMC208823 14504398

[74] Bhattacharya S, Ray RM, Johnson LR. 2005. Decreased apoptosis in polyamine depleted IEC-6 cells depends on Akt-mediated NF-κB activation but not GSK3β activity. Apoptosis 10:759–776. doi:10.1007/s10495-005-2943-3 16133867

[75] Kucharzewska P, Welch JE, Svensson KJ, Belting M. 2009. The polyamines regulate endothelial cell survival during hypoxic stress through PI3K/AKT and MCL-1. Biochem Biophys Res Commun 380:413–418. doi:10.1016/j.bbrc.2009.01.097 19250631

[76] Bratton DL, Fadok VA, Richter DA, Kailey JM, Frasch SC, Nakamura T, Henson PM. 1999. Polyamine regulation of plasma membrane phospholipid flip-flop during apoptosis. J Biol Chem 274:28113–28120. doi:10.1074/jbc.274.40.28113 10497162

[77] Zhao C, Ma Y, Zhang M, Gao X, Liang W, Qin Y, Fu Y, Jia M, Song H, Gao C, Zhao W. 2023. Polyamine metabolism controls B-to-Z DNA transition to orchestrate DNA sensor cGAS activity. Immunity 56:2508–2522. doi:10.1016/j.immuni.2023.09.012 37848037

[78] Baldridge MT, Nice TJ, McCune BT, Yokoyama CC, Kambal A, Wheadon M, Diamond MS, Ivanova Y, Artyomov M, Virgin HW. 2015. Commensal microbes and interferon-λ determine persistence of enteric murine norovirus infection. Science 347:266–269. doi:10.1126/science.1258025 PMC4409937 25431490

[79] Michael AJ. 2018. Polyamine function in archaea and bacteria. J Biol Chem 293:18693–18701. doi:10.1074/jbc.TM118.005670 PMC6290158 30254075

[80] Yu L, Zhang C, Zhai Q. 2024. Gut microbes participate in host polyamine metabolism. Proc Natl Acad Sci USA 121:e2419368121. doi:10.1073/pnas.2419368121 PMC11551406 39467143

[81] Karst SM, Wobus CE. 2015. A working model of how noroviruses infect the intestine. PLoS Pathog 11:e1004626. doi:10.1371/journal.ppat.1004626 PMC4344369 25723501

[82] Peiper AM, Morales Aparicio J, Hu Z, Phophi L, Helm EW, Rubinstein RJ, Phillips M, Williams CG, Subramanian S, Cross M, Iyer N, Nguyen Q, Newsome R, Jobin C, Langel SN, Bucardo F, Becker-Dreps S, Tan XD, Dawson PA, Karst SM. 2024. Metabolic immaturity and breastmilk bile acid metabolites are central determinants of heightened newborn vulnerability to norovirus diarrhea. Cell Host Microbe 32:1488–1501. doi:10.1016/j.chom.2024.08.003 PMC11392616 39214086

[83] Steverding D. 2010. The development of drugs for treatment of sleeping sickness: a historical review. Parasit Vectors 3:15. doi:10.1186/1756-3305-3-15 PMC2848007 20219092

[84] Kuemmerle A, Schmid C, Bernhard S, Kande V, Mutombo W, Ilunga M, Lumpungu I, Mutanda S, Nganzobo P, Tete DN, Kisala M, Burri C, Blesson S, Valverde Mordt O. 2021. Effectiveness of Nifurtimox Eflornithine Combination Therapy (NECT) in *T. b. gambiense* second stage sleeping sickness patients in the Democratic Republic of Congo: report from a field study. PLoS Negl Trop Dis 15:e0009903. doi:10.1371/journal.pntd.0009903 PMC8601604 34748572

[85] Goyal L, Supko JG, Berlin J, Blaszkowsky LS, Carpenter A, Heuman DM, Hilderbrand SL, Stuart KE, Cotler S, Senzer NN, Chan E, Berg CL, Clark JW, Hezel AF, Ryan DP, Zhu AX. 2013. Phase 1 study of *N*^1^,*N*^11^-diethylnorspermine (DENSPM) in patients with advanced hepatocellular carcinoma. Cancer Chemother Pharmacol 72:1305–1314. doi:10.1007/s00280-013-2293-8 24121453

[86] Wolff AC, Armstrong DK, Fetting JH, Carducci MK, Riley CD, Bender JF, Casero RA Jr, Davidson NE. 2003. A phase II study of the polyamine analog *N*^1^,*N*^11^-diethylnorspermine (DENSpm) daily for five days every 21 days in patients with previously treated metastatic breast cancer. Clin Cancer Res 9:5922–5928. 14676116

[87] Wolff AC, Bowling MK, DeClue C, Armstrong DK, Fetting JH, Casero Jr RA, Davidson NE. 2001. A phase I study of diethylnorspermine (DENSPM) in previously treated patients with metastatic breast cancer (MBC). Breast Cancer Res Treat 69:286.

[88] Larqué E, Sabater-Molina M, Zamora S. 2007. Biological significance of dietary polyamines. Nutrition 23:87–95. doi:10.1016/j.nut.2006.09.006 17113752

